# Mechanical Durability of Polymer-Encapsulated Electronic Yarns for Electronic Textile Applications †

**DOI:** 10.3390/polym18151923

**Published:** 2026-08-05

**Authors:** Tharushi Peiris, Lukas Werft, Sigrid Rotzler, Arash M. Shahidi, Kalana Marasinghe, Carlos Oliveira, Tilak Dias, Theo Hughes-Riley

**Affiliations:** 1 Advanced Textiles Research Group, Nottingham School of Art and Design, Nottingham Trent University, Nottingham NG7 4HF, UK; thelge.peiris2023@my.ntu.ac.uk (T.P.); arash.shahidi@ntu.ac.uk (A.M.S.); kalana.marasinghe2025@my.ntu.ac.uk (K.M.); jose.oliveira@ntu.ac.uk (C.O.); tilak.dias@ntu.ac.uk (T.D.); 2 Fraunhofer Institute for Reliability and Microintegration IZM, 13355 Berlin, Germany; lukas.werft@izm.fraunhofer.de

**Keywords:** electronic textiles, E-textiles, smart textiles, electronic yarns, wearables, polymer encapsulation, mechanical reliability, bending fatigue, torsional fatigue, tensile loading, wash durability

## Abstract

This study presents a standalone yarn-level assessment of the mechanical and functional durability of polymer-encapsulated electronic yarns (E-yarns) for wearable electronic textile applications. The investigated E-yarns consisted of miniaturised electronic components soldered onto fine conductive wires, protected by polymer encapsulation, and enclosed within braided textile yarn structures. This heterogeneous architecture enables textile-compatible functionality but creates local regions that may be susceptible to damage under various deformation modes. E-yarns incorporating light-emitting diode, photodiode, and resistor components were evaluated under cyclic bending fatigue, torsional fatigue, quasi-static tensile loading, and wash durability conditions. Electrical measurements were used to monitor functional degradation and failure, while X-ray imaging, scanning electron microscopy and finite element analysis were used to examine structural damage, failure localisation, fracture surface morphology, and local stress and strain distribution. The results show that E-yarn durability depended on the imposed loading condition, with distinct mechanical and functional responses observed across the different test modes. The polymer-encapsulated region emerged as a mechanically important feature of the E-yarn architecture, particularly at transitions between encapsulated and non-encapsulated regions. By addressing multiple deformation and loading conditions at the standalone yarn level, this work provides a systematic reliability assessment of polymer-encapsulated E-yarns, enabling intrinsic failure mechanisms to be distinguished from textile integration effects and supporting the development of more reliable yarn-based electronic textiles.

## 1. Introduction

This article focuses on the mechanical durability testing of a specific type of electronic textile (E-textile), electronic yarns (E-yarns), and seeks to understand how design decisions affect durability while presenting a comprehensive methodology for mechanically testing yarn-based electronics. This work builds on findings presented at the E-textiles 2025 Conference [1] (the associated proceeding is not currently publicly available), adding substantial experimental data, statistical analysis and a more detailed assessment of the observed reliability behaviour. This extended analysis provides a more robust basis for identifying which influencing factors are statistically supported and which should be interpreted as preliminary observations.

The goal of many wearable E-textiles is to create a material architecture that combines electronic functionality with the compliance, flexibility, and care requirements of conventional textiles, thereby producing a system that is comfortable for the wearer and durable. In these systems, polymers are often used as encapsulants, coatings, substrates or interfacial materials to protect electronic components and conductors, enabling their integration with soft textile structures [2]. The polymeric regions can strongly influence mechanical stability, environmental resistance, strain transfer, and failure behaviour during real-world use [3,4]. For wearable E-textiles, understanding the durability of these polymer-supported electronic structures is essential, particularly when they are exposed to repeated deformation due to wear and use conditions.

E-yarns provide a yarn-level approach for embedding electronic functionality within textile structures. This approach integrates miniaturised components directly within a yarn format. To construct an E-yarn, a miniaturised electronic component is first soldered onto fine conductive wires and combined with supporting yarns before being encapsulated within a polymer micro-pod. This encapsulated region protects the component and solder joints from mechanical and environmental exposure and provides local structural support. The assembly is then enclosed within a braided textile sheath to consolidate the structure and improve textile compatibility and handling. This patented technique has been employed to develop wearable E-textile applications, including healthcare monitoring, wellbeing, protection, illumination, and interactive textiles [5,6]. For example, near-fall detection socks incorporating IMU based E-yarns have been developed to support older adults, alongside temperature sensing socks using embedded thermal sensor yarns to provide thermoregulation [5]. A vibrotactile glove capable of providing haptic feedback has also been realised using this core E-yarn technology [5]. Different embedded components and applications will result in E-yarns with different internal and external architectures [7]. While this technique enables the electronic component to be incorporated into a robust yarn-like structure, it also creates a complex hybrid system consisting of rigid, semirigid, and flexible elements. As a result, the polymer micro-pod becomes a critical region where protection, stiffness transition, and stress transfer are simultaneously controlled.

Due to its heterogeneous nature, the mechanical response of an E-yarn is expected to differ from that of a conventional yarn, thereby affecting durability and points of failure. The presence of a rigid component, soldered wire joints, polymer encapsulation, conductive wires, supporting yarns, and a braid covering creates local changes in geometry and stiffness along the length of the yarn. These discontinuities can influence the way strain is distributed during bending, twisting, tensile loading, and handling. Textile braiding further modifies the structural behaviour by introducing specific geometrical and mechanical characteristics that depend on braiding material, braid angle, yarn coverage, and construction parameters [8]. Consequently, the wiring configuration, the design of the encapsulated region, and the surrounding textile sheath are likely to significantly influence both the mechanical resilience and the electrical stability of the E-yarn.

Following fabrication, E-yarns can be integrated into textile assemblies through established construction techniques, with weaving, incorporation within knitted channels as a post-process, or couching most commonly used [9].

Durability remains a major limitation in the translation of many E-textiles from prototype demonstrations to practical wearable products, and as such there has been significant academic interest in this area [10,11,12]. During use, textile-based electronic systems may experience repeated axial tension, bending, torsion, compression, abrasion, moisture exposure, and laundering. Textile durability testing is a mature field with various established standards for the mechanical evaluation of textile products and garments [13,14,15,16]. The hybrid electronic structures of E-textiles introduce additional considerations as mechanical, electrical, and functional failures can all occur [4,17,18,19]. This behaviour is largely attributed to the heterogeneous nature of most E-textiles, where the coexistence of materials with differing mechanical properties introduces localised stress concentrations, such that failure preferentially occurs at mechanically vulnerable regions including interconnects, interfaces, and transitions between materials of varying stiffness. Studies on flexible and stretchable electronics have shown that mechanical mismatch, repeated strain, and interfacial damage can lead to electrical degradation, conductor fracture, and functional failure [20,21]. Similar failure principles are relevant to E-yarns, but the yarn architecture introduces additional complexity as the conductive pathway and embedded component are surrounded by polymer encapsulation and textile covering.

Although many yarn and fibre-based E-textile innovations have been reported [22], the methods of mechanically testing the durability of these are not standardised, which is also true in the wider E-textile literature [10,23,24]. Recent wearable electronic yarn research has continued to focus on improving the durability and reliability of yarn-based electronic systems through advances in sensing yarn architectures, textile integration approaches, and encapsulation strategies [9,25,26,27]. These studies demonstrate the increasing maturity of wearable electronic yarn technologies. However, durability assessment methodologies remain diverse and application-dependent, making direct comparison between studies challenging. Testing reported in the literature is also seldom comprehensive and is rarely the primary focus of the work, often limiting detailed analysis of failure mechanisms.

Chen et al. reported detailed yarn-level testing protocols including electromechanical characterisation of three classes of conductive yarns used in E-textiles, metal-based, metal-coated, and polymer-based yarns, under tensile and bending loading [28]. Their work demonstrated that deformation-induced resistance changes are strongly dependent on yarn material and structure. Similar investigations of silver-plated and polymer-based conductive yarns used as interconnects in E-textiles subjected these yarns to repeated mechanical stresses, while electrical resistance was measured during loading to assess durability [29]. In addition, studies examining conductive yarn elements integrated for data transmission have applied tensile loading with simultaneous electrical measurements to evaluate electromechanical coupling under strain [30]. Furthermore, testing frameworks developed for flexible and wearable electronics apply combined bending, stretching, and twisting deformation modes with in situ resistance monitoring, demonstrating that electrical degradation depends on the type and sequence of applied mechanical loading [31]. However, these studies predominantly investigate relatively homogeneous conductive yarn systems, where the conductive pathway is continuous (e.g., metal yarns), coated (e.g., silver-plated yarns), or uniformly distributed within polymer matrices [28,29], and therefore do not capture the behaviour of structurally heterogeneous yarns used in E-textile developments. For E-yarns, some mechanical testing has been presented, including tensile tests [7]; however, a comprehensive regime of mechanical tests using different architectures had not been presented prior to the authors’ study [1].

Furthermore, most of the published durability literature evaluates performance metrics such as resistance change, survivability, or post-test functionality [17,18,28,30,31]. However, relatively few studies examine the mechanical origins of degradation, such as stiffness mismatch, interfacial damage, conductor fracture, and localised strain accumulation [4,17,18,19,21]. For polymer-encapsulated E-yarns, the interaction between rigid electronic components, solder interconnections, conductive wires, polymer micro-pods, and textile reinforcement introduces multiple potential failure pathways that cannot be inferred directly from conventional conductive yarn studies. Consequently, although previous work has demonstrated the feasibility and durability of E-yarn technologies, a mechanistic understanding of how failure initiates and propagates within the standalone E-yarn architecture remains limited.

Laundering is also an important durability condition for wearable E-textiles, as wash durability is a basic requirement for garments and textile-based products. Recent work on textile integrated circuits has shown that washing behaviour depends on the textile substrate, circuit material, and integration method [23]. Although standardised wash testing protocols for E-textiles have recently been developed [32], these are not consistently adopted in academic research, with many now employing a variation on standard textile washing protocols as highlighted in [23], whereas others still use less robust tests. For E-yarns, encapsulation size and textile integration technique have also been shown to influence wash durability in textiles containing integrated E-yarns [9]. Hardy et al. investigated the wash testing of electronic yarns and reported that E-yarn architectures can retain functionality under laundering, although failures were associated with factors such as wire damage and micro-pod related limitations [33]. While these studies provide important evidence for the washability of E-yarn-based systems, the yarns are commonly evaluated after being integrated into, attached to, or supported by textile structures. As a result, the intrinsic wash durability of free-standing polymer-encapsulated E-yarns has not been clearly isolated. This distinction is important because textile integration can either protect or mechanically constrain an E-yarn during washing and deformation. A surrounding fabric may reduce direct exposure to agitation, change the bending radius experienced by the yarn, or introduce additional stresses depending on the integration technique. Therefore, testing only after textile integration does not fully determine whether the failure originates from the E-yarn architecture itself or from the combined yarn and textile assembly. Direct yarn-level testing is required to evaluate the fundamental reliability of the encapsulated component region, conductive interconnections, polymer micro-pod, and braided covering before the yarn is incorporated into more complex textile platforms.

Despite the growing body of work demonstrating E-yarn functionality and wash durability, a fundamental reliability gap remains. Previous studies have predominantly evaluated E-yarns after textile integration or under application-specific durability conditions, particularly laundering, where failures have been observed but cannot be unequivocally attributed to the E-yarn architecture itself because the surrounding textile structure alters the mechanical environment experienced by the yarn [9,33]. Similarly, existing E-yarn investigations have largely focused on design development or isolated loading scenarios [7], while durability studies on conductive yarns have primarily examined more homogeneous conductive structures rather than heterogeneous yarn systems containing embedded electronic components [28,29,30]. Consequently, the underlying failure mechanisms of standalone polymer-encapsulated E-yarns remain poorly understood. In particular, the influence of different deformation modes on damage accumulation, failure localisation, and functional degradation has not been systematically investigated at the yarn level. This lack of fundamental reliability data limits the development of design guidelines and prevents clear distinction between failures originating from the E-yarn itself and those introduced through textile integration.

The present study investigates the mechanical and functional durability of polymer -encapsulated multifunctional E-yarns for wearable textile applications by examining five discrete yarn configurations. Light-emitting diode (LED), photodiode (PD), and resistor E-yarns were selected to represent different component geometries and functional roles. The E-yarns were evaluated under four key testing conditions: cyclic bending fatigue, torsional fatigue, quasi-static tensile loading, and direct wash exposure applied to free-standing yarn specimens. Electrical performance was monitored to identify functional degradation and failure, and post-failure characterisation was conducted using scanning electron microscopy and X-ray imaging to examine structural damage, localise failure, and analyse fracture surfaces. By focusing on the E-yarn before textile integration, this study provides a systematic standalone reliability assessment of polymer-encapsulated multifunctional E-yarns across multiple fundamental deformation modes. The novelty of this work lies in combining cyclic bending fatigue, torsional fatigue, quasi-static tensile loading, and direct wash exposure with continuous electrical functionality monitoring and post-failure structural characterisation using X-ray imaging and scanning electron microscopy. This integrated methodology enables intrinsic failure mechanisms to be identified and distinguished from textile-level effects that may obscure damage evolution once E-yarns are incorporated into fabrics. The resulting mechanistic understanding of the role of component architecture, polymer encapsulation, conductive interconnections, and braided reinforcement provides new knowledge for the design of more reliable E-yarns and supports the development of durability assessment methodologies for future yarn-based electronic textiles.

## 2. Materials and Methods

### 2.1. E-Yarn Fabrication

#### 2.1.1. Core E-Yarn Architecture

**Figure 1:**
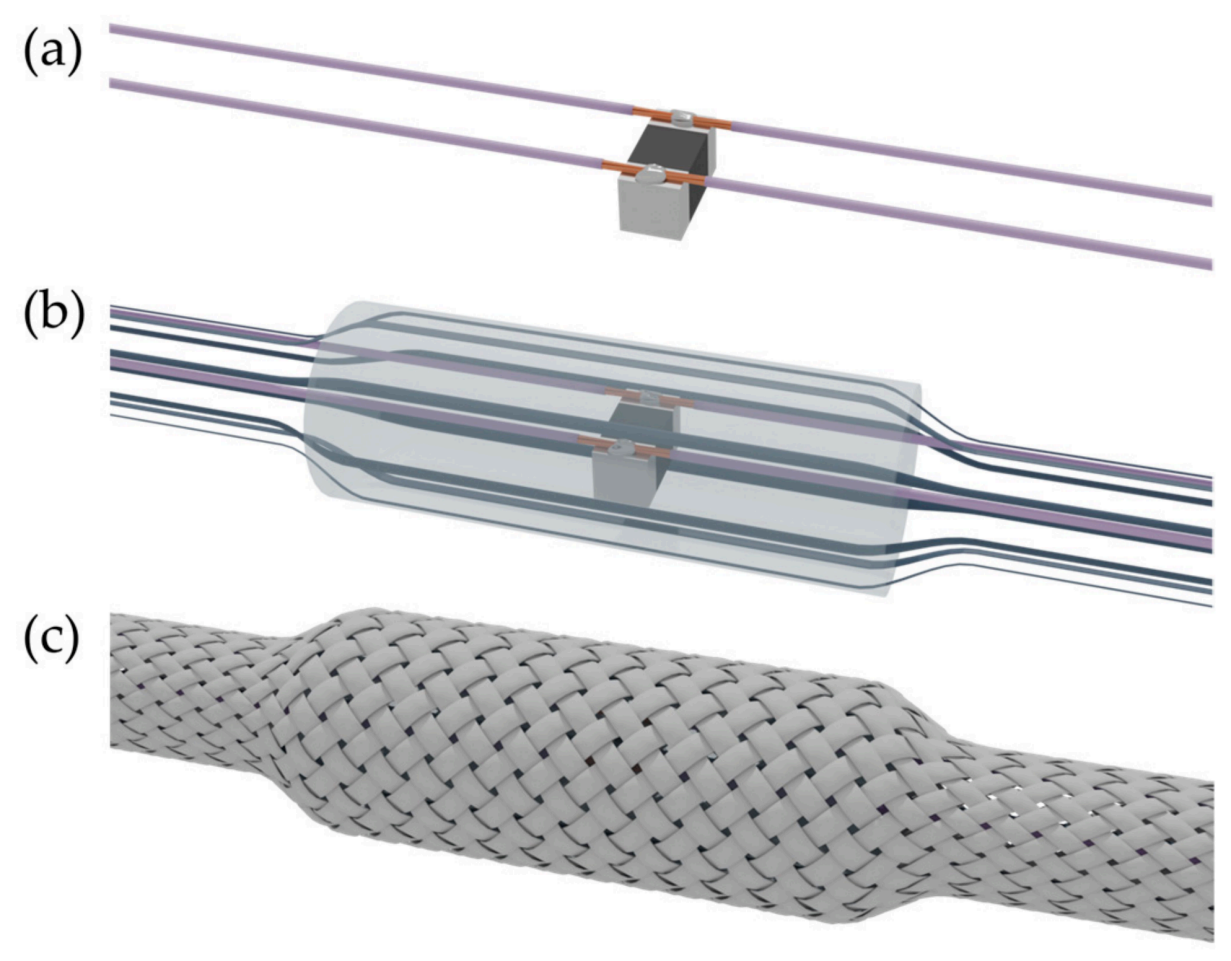
Schematic of core E-yarn fabrication stages. (**a**) Soldering of the electronic component onto conductive Litz wires; (**b**) polymer micro-pod encapsulation with the addition of supporting yarn(s); (**c**) textile braiding of the encapsulated yarn structure.

The core E-yarn manufacturing process consists of three main fabrication stages, including soldering, polymer encapsulation and textile braiding, as illustrated in Figure 1.

During the soldering stage, miniaturised electronic components are soldered onto commercially available conductive wires (Litz wires; BXL2001-Type 1, OSCO Ltd., Milton Keynes, UK). These wires comprise a multistrand (seven strands) core conductor made of twisted copper (Cu) strands, insulated with an enamel coating and further wrapped with a textile fibre covering. Soldering is generally performed manually using a soldering iron (AX25, Antex Electronics, Plymouth, UK) and lead-free solder wire (RS PRO wire, o.d. = 0.25 mm, melting point 228 °C; RS Components Ltd., Northants, UK).

Following soldering, the components were encapsulated with a polymer, forming a micro-pod around the component and solder joints, creating a robust yarn structure, designed to minimise environmental exposure and mechanical damage. A commercially available UV-curable polymer (Dymax 9001-E-V3.5; Dymax Corporation, Torrington, CT, USA) was used for all samples in this study. To ensure consistent pod geometry and mechanical integrity, a silicone mould guide was employed. The components soldered onto Litz wires were positioned centrally within the mould, while the remaining lengths of the yarn extended freely beyond the mould. In addition, to enhance the tensile strength of the final E-yarn and improve stress distribution along the yarn axis, one or more supporting yarns were added and aligned parallel to the longitudinal axis of the E-yarn. The supporting yarn material and number were selected based on the intended application of the E-yarn and are described below.

The polymer was injected into the mould, fully enclosing the electronic component and solder joints. The encapsulant was subsequently cured under ultraviolet (UV) illumination (Dymax BlueWave^®^ QX4™, Dymax Corporation) for 60 s, creating a robust structure.

These structures with encapsulated electronic components, Litz wires, and supporting yarns were then covered with a tubular textile braid structure using a rotary textile braiding machine (Herzog RU 1/24-80; HERZOG GmbH, Oldenburg, Germany). Both braid covering material and braiding parameters were varied according to the specific E-yarn application requirements and are described in detail below for each E-yarn used in this work.

#### 2.1.2. E-Yarn Configurations

**Figure 2:**
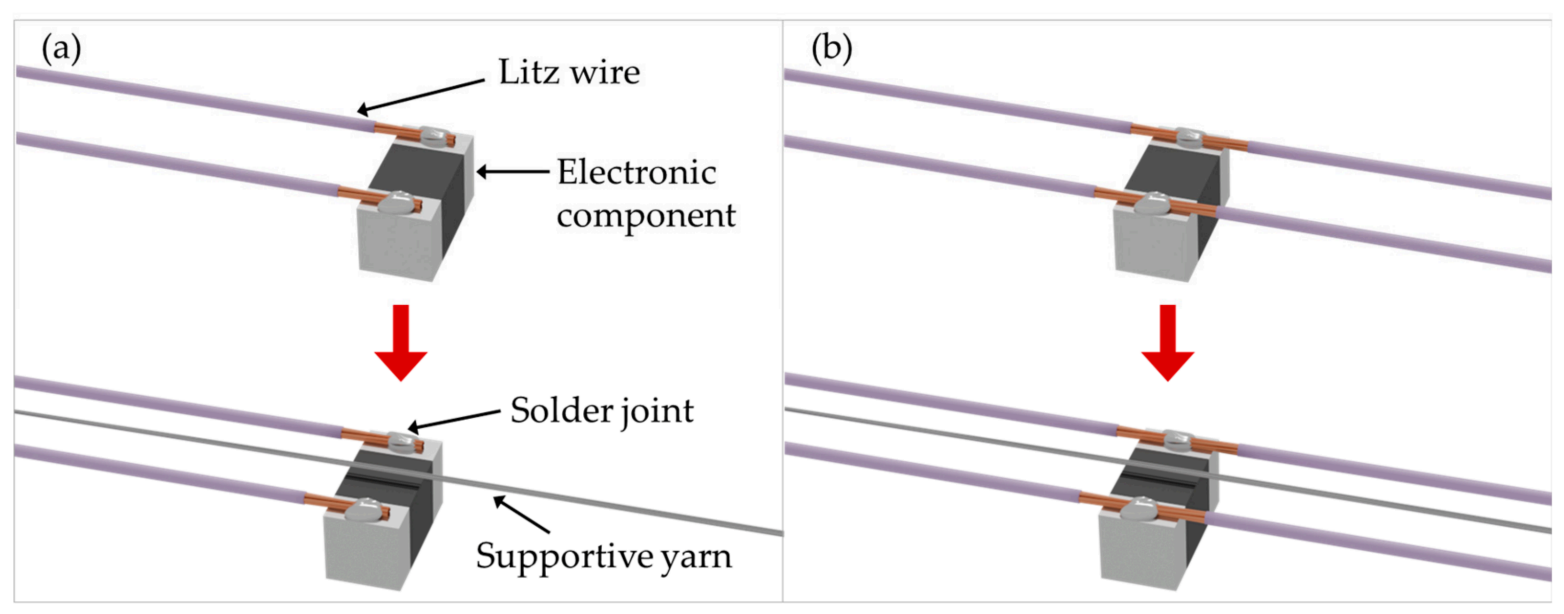
Schematic representation of E-yarn wiring configurations along with component positioning. (**a**) End-soldered E-yarn configuration; (**b**) mid-soldered E-yarn configuration. Red arrows indicate the addition of the Vectran yarns following soldering.

E-yarns can employ different wiring configurations depending on the intended application and component arrangement. This study focused on two commonly used configurations. In the first configuration, referred to here as the end-soldered (ES) configuration, the electronic component is soldered onto the ends of the conductive Litz wires, causing the wires to terminate at the embedded component. This arrangement is typically used for two-terminal sensing elements. In the second configuration, referred to as the mid-soldered (MS) configuration, the electronic component is soldered at the midpoint of the Litz wires, allowing the conductive wires to extend along the yarn length on both sides of the component. This arrangement is commonly used in LED-based E-yarns. To investigate the influence of solder wiring configuration and structural symmetry on E-yarn reliability, these two component positioning approaches were explored, as illustrated in Figure 2.

For this study a range of common miniature surface mount electronic components were employed including light emitting diodes (LEDs; 1.0 mm × 0.5 mm × 0.5 mm; KPHHS-1005SURCK, Kingbright Electronic Co. Ltd., New Taipei City, Taiwan), photodiodes (PDs; 4.0 mm × 2.0 mm × 1.05 mm; VEMD 6060X01, Vishay Intertechnology Inc., Shelton, CT, USA), and resistors (1.0 mm × 0.5 mm × 0.35 mm; 10 kΩ, CRCW040210K0FKED, Vishay Intertechnology Inc.). The components were soldered onto the fine Litz wires with the ES configuration. In addition, a subset of LEDs was soldered with the MS configuration. The resistor-embedded E-yarns represented standard two-terminal electronic components commonly used in E-yarn-based E-textile developments and were therefore selected as reference samples for general-purpose applications. The LED and PD yarns represent a standard E-yarn type, with the specific configuration chosen to represent LED and PD E-yarns used for optical sensing and physiological signal acquisition through photoplethysmography, as demonstrated in previous studies [6].

Two polymer encapsulation pod geometries were explored in this study. Cuboid micro-pods were used for the LED and PD E-yarns, while cylindrical micro-pods were employed for the resistor E-yarns, reflecting the standard pod geometry typically used in conventional E-yarn fabrication (specifications detailed in Table 1).

Vectran™ yarn (Kuraray America Inc., Tokyo, Japan) was used as the supporting yarn in all samples, aiding the encapsulation process and providing additional tensile reinforcement to the final E-yarn structure.

To isolate and understand the mechanical and structural behaviour of the Litz wires in the absence of soldering and polymer encapsulation, an additional set of control samples was fabricated. These samples comprised two Litz wires and one Vectran™ yarn, corresponding to the key core yarn constituents common to all other E-yarn configurations used in this study. The three yarns were aligned in parallel and braided together using the same baseline braid covering material and braiding parameters employed for the encapsulated E-yarns, resulting in a comparable tubular braided E-yarn structure. These control samples enabled a direct assessment of the influence of soldering and encapsulation on yarn behaviour by providing a reference structure with an identical core yarn composition and braid configuration but without embedded electronic components or polymer micro-pods.

The primary focus of this work was to understand the influence of the polymer-encapsulated component region on the mechanical durability of E-yarns. Therefore, the yarns were completed using their predefined application-specific braiding configurations rather than varying braid architecture as an independent experimental parameter. The braid covering yarns and lay length used for the LED and PD E-yarns were defined according to the required coverage, compactness, and structural integrity of their intended application-specific designs, and the same design parameters were retained for the samples tested in this study. This design was identical to that used in earlier work [6]. These E-yarns were produced using a 12-carrier braid structure and a 10 mm lay length. For the resistor E-yarns, a previously established general E-yarn design was replicated without modification to provide a reference configuration [7]. This reference design has been used in other durability studies (tensile strength [7], wash durability [9]), providing a direct comparison for this study. This design used a 24-carrier braid structure with a 6 mm lay length. Polyester yarns (J. H. Ashworth & Son Ltd., Hyde, UK) were used for all braid coverings. The authors acknowledge that braid construction can influence durability through yarn confinement, compactness and load sharing. However, in this initial standalone E-yarn durability study, braid architecture was retained as part of the defined E-yarn design configuration rather than varied independently. Future work should systematically investigate the influence of braid structure on E-yarn failure behaviour.

The specifications of the E-yarn samples fabricated for this study, including the E-yarn configuration, encapsulation micro-pod geometry and braiding specifications, are summarised in Table 1. Thicknesses of the braided E-yarns in the non-encapsulated region, presented in Table 1, were measured using a digital calliper (Farnell Multicomp Pro, Leeds, UK).

**Figure 3:**
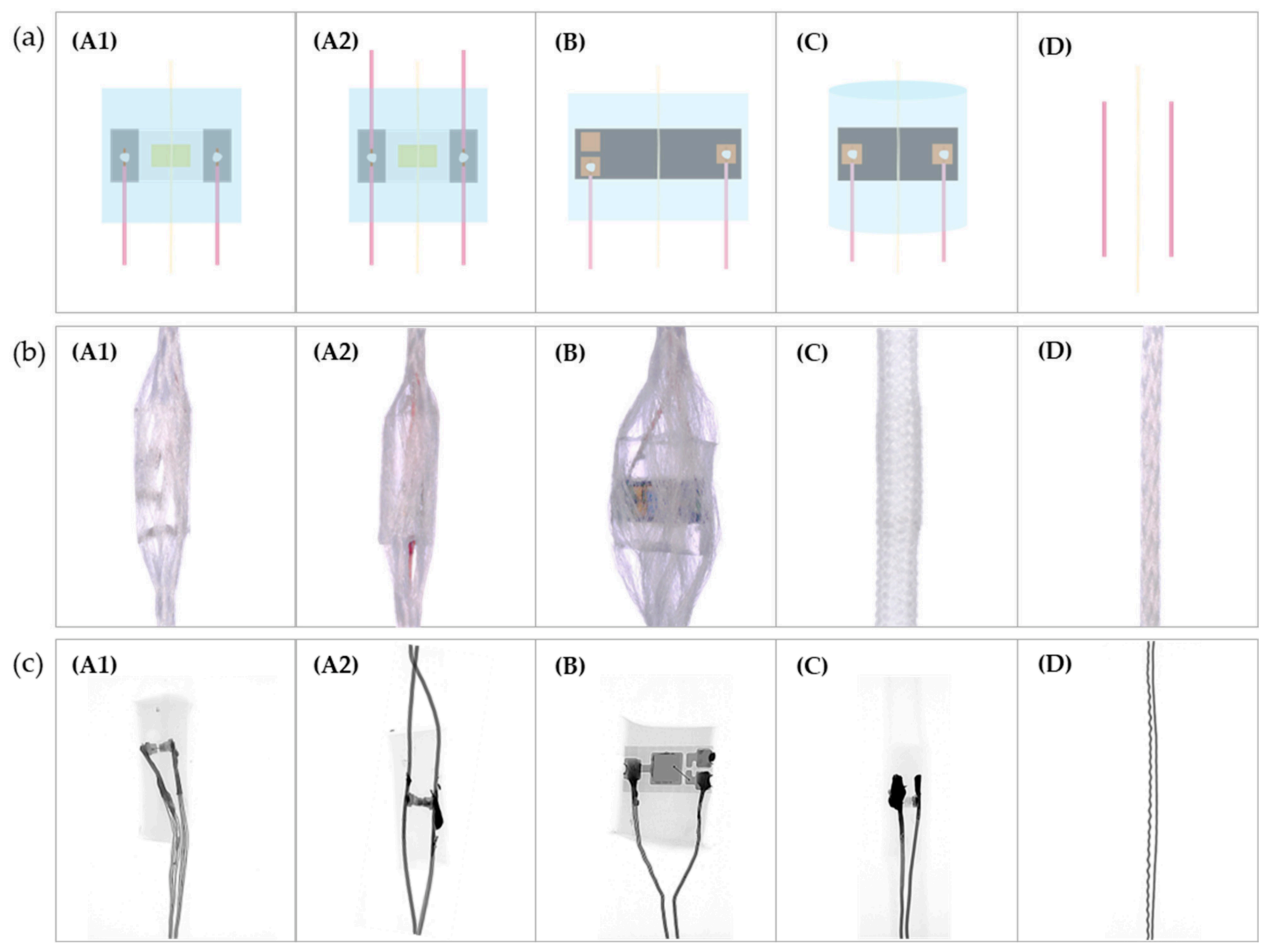
Schematic and experimental visualisation of the E-yarn configurations. (**a**) Schematic of E-yarn structures prior to braiding; (**b**) representative microscopic images of the final E-yarns; (**c**) representative X-ray images of each E-yarn configuration. (**A1**) End-soldered LED E-yarn. (**A2**) Mid-soldered LED E-yarn. (**B**) End-soldered PD E-yarn. (**C**) End-soldered resistor E-yarn. (**D**) Braided Litz wires.

Representative images of the E-yarn configurations used in this study are presented in Figure 3.

### 2.2. Mechanical Durability Experimental Protocol

To evaluate the mechanical reliability of the E-yarns in their standalone yarn form, a series of mechanical durability tests was conducted. These comprised two cyclic fatigue tests (bending and torsional fatigue), a quasi-static tensile loading test and wash durability tests. The selection of loading modes and experimental designs was based on replicating the dominant deformation conditions experienced by E-yarns in wearable applications and to allow for the primary failure mode to be determined. Accordingly, bending fatigue and torsional fatigue tests were performed to assess cyclic durability, alongside quasi-static tensile loading and wash durability tests to evaluate residual mechanical robustness.

Bending and torsional fatigue experiments were conducted at Fraunhofer IZM (Fraunhofer Institute for Reliability and Microintegration IZM, Berlin, Germany) while tensile loading and wash durability tests were carried out at Nottingham Trent University (Nottingham, UK). The experimental protocols adopted for bending fatigue, torsional fatigue, and tensile loading were previously reported in [1].

Five specimens were selected for each tested E-yarn configuration as a baseline sample size for the comparative durability assessments, in line with published E-textile studies that have used similar specimen numbers for mechanical, electromechanical, bending, and wash durability characterisations [28,34,35].

#### 2.2.1. Bending Fatigue Test

Bending fatigue tests were conducted using an Instron ElectroPuls E10000 Linear-Torsion all-electric dynamic tester (Instron^®^, Norwood, MA, USA), configured to apply both cyclic linear and rotational loading. This testing apparatus has been previously used for the mechanical characterisation of flexible and stretchable electronic systems [21].

The bending fatigue experiment was designed to cyclically deform the E-yarn between a specified bending angle and a corresponding recovery angle. The bending angle quantifies the angular separation between the yarn segments during bending deformation, while the recovery angle describes the extent of realignment toward the original yarn positioning. Custom-designed bending arms were mounted on the Instron system’s original clamps to enable bending movement, with the angular positions synchronised to the linear displacements of the original clamps.

In this test configuration, the lower clamp remained stationary, while the upper clamp was driven using a sinusoidal displacement profile. The displacement limits were first defined from the clamp positions corresponding to the recovery and maximum bent states of the yarn. The displacement amplitude was then calculated as shown in Equation (1) and used to define the sinusoidal motion applied during cyclic bending.

(1) $$A=\frac{\;l_{\mathrm{Recovery}}-l_{\mathrm{Bent}\;}\;}{2}$$

where *A* is the linear displacement amplitude, *l_Recovery_* is the vertical position of the upper clamp corresponding to the recovery state of the yarn, and *l_Bent_* is the vertical position of the upper clamp corresponding to the maximum bent state.

**Figure 4:**
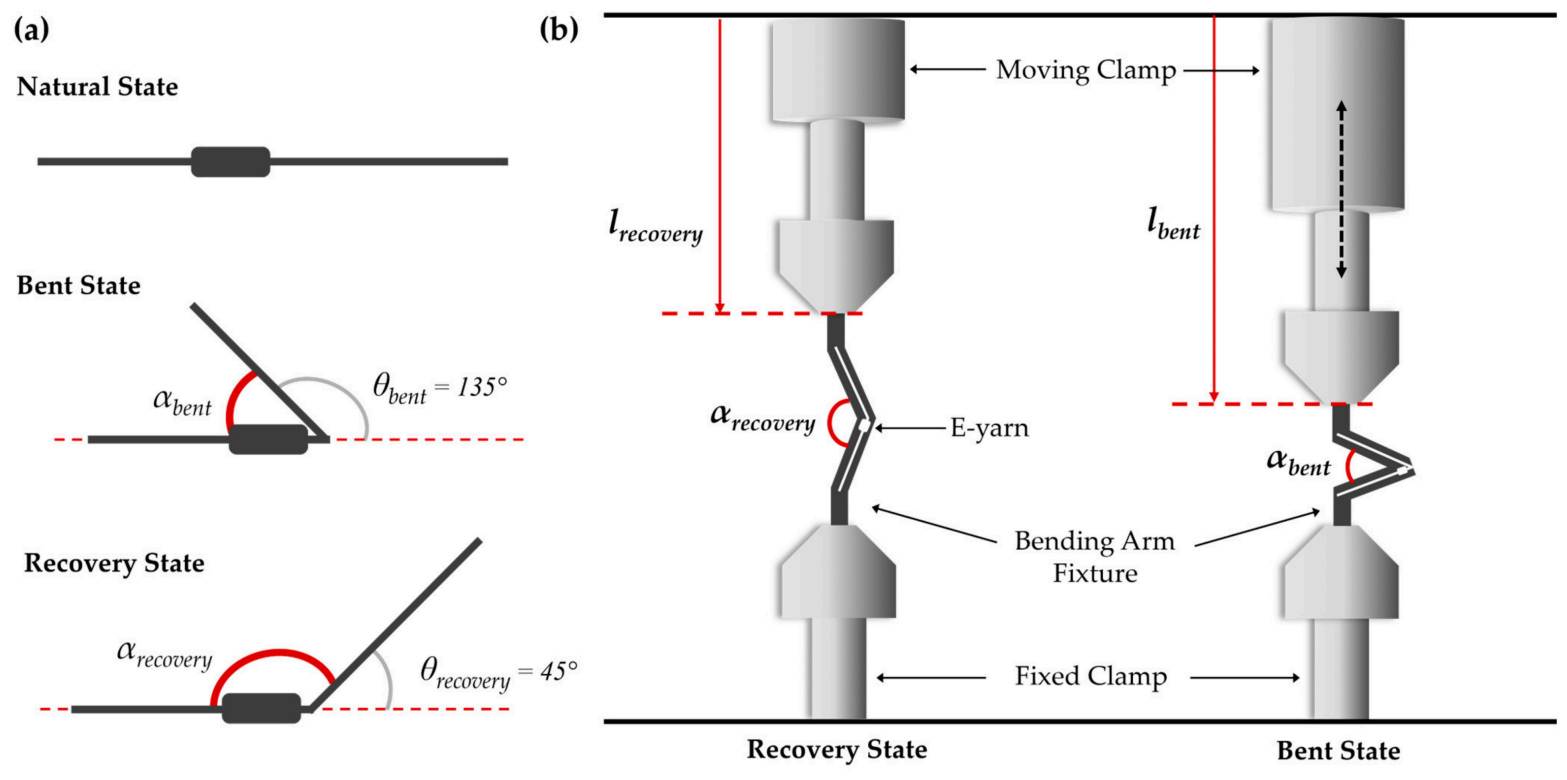
Schematic representation of the bending fatigue test configuration, illustrating the recovery and bent states of the E-yarn during cyclic loading. (**a**) Angular deformations in the natural state, bent state and recovery state of the E-yarn; (**b**) clamp positioning and yarn fixture in the bending test setup.

A maximum bent state of 45° angle between the two yarn segments was selected for cyclic bending fatigue testing. This corresponds to an angular bending deformation of 135° from the straight yarn condition, as illustrated in Figure 4a. This loading condition was selected to impose a severe and repeatable yarn-level folding deformation, exceeding the 90° bending conditions commonly reported in E-textile-related studies [36,37,38]. The recovery state was defined at an angle of 135°, corresponding to an angular bending deformation of 45° from the straight yarn condition. Although the testing system was intrinsically capable of complete angular recovery of up to 180°, this configuration could not be implemented due to mechanical restrictions imposed by the custom bending arm fixture and yarn clamping constraints. The selected bending condition was therefore used as a demanding comparative laboratory test for assessing E-yarn durability under high bending deformation, rather than as a direct simulation of a single standardised biomechanical movement.

The angle formed between the yarn segments is denoted by *α*, as illustrated in Figure 4b. For bending strain analysis, the corresponding curvature angle (*θ*) is defined as

(2) θ =π−α,

Which can be applied in the fundamental bending strain (*ε*) equation describing the maximum tensile or compressive surface deformation in the E-yarn structure

(3) $$\varepsilon =\frac{t}{2\;R}=\frac{\;t\;\theta \;}{2\;L}$$

where *t* is the E-yarn thickness, *R* is the bending radius, and *L* is the arc length of the bent section.

The bending region in this study was positioned at the interface between the encapsulated region and the standard yarn segment, as this location is most susceptible to mechanically induced failure. Previous investigations of wash durability reported in [33] have shown that this transition point consistently acts as the primary failure location. This vulnerability arises from structural discontinuities such as soldered connections, embedded rigid components and the interface between encapsulated and non-encapsulated yarn regions, all of which create localised weaknesses within the E-yarn structure.

The E-yarn specimens were positioned in an asymmetric arrangement between the two clamping fixtures. For the samples with the ES configuration, the section of the E-yarn containing the conductive Litz wires was attached to the oscillating bending arm using non-conductive adhesive tape, ensuring mechanical stability while preserving electrical continuity. Electrical connections for real-time monitoring were established by attaching measurement clips directly to the exposed ends of the Litz wires. The opposite section of the E-yarn, which did not incorporate conductive elements, was passed through a heat shrink tube and secured to the stationary bending arm. To minimise axial slippage and to maintain consistent angular movement during bending, a weight of 20 mN was added to this non-conductive yarn section.

**Figure 5:**
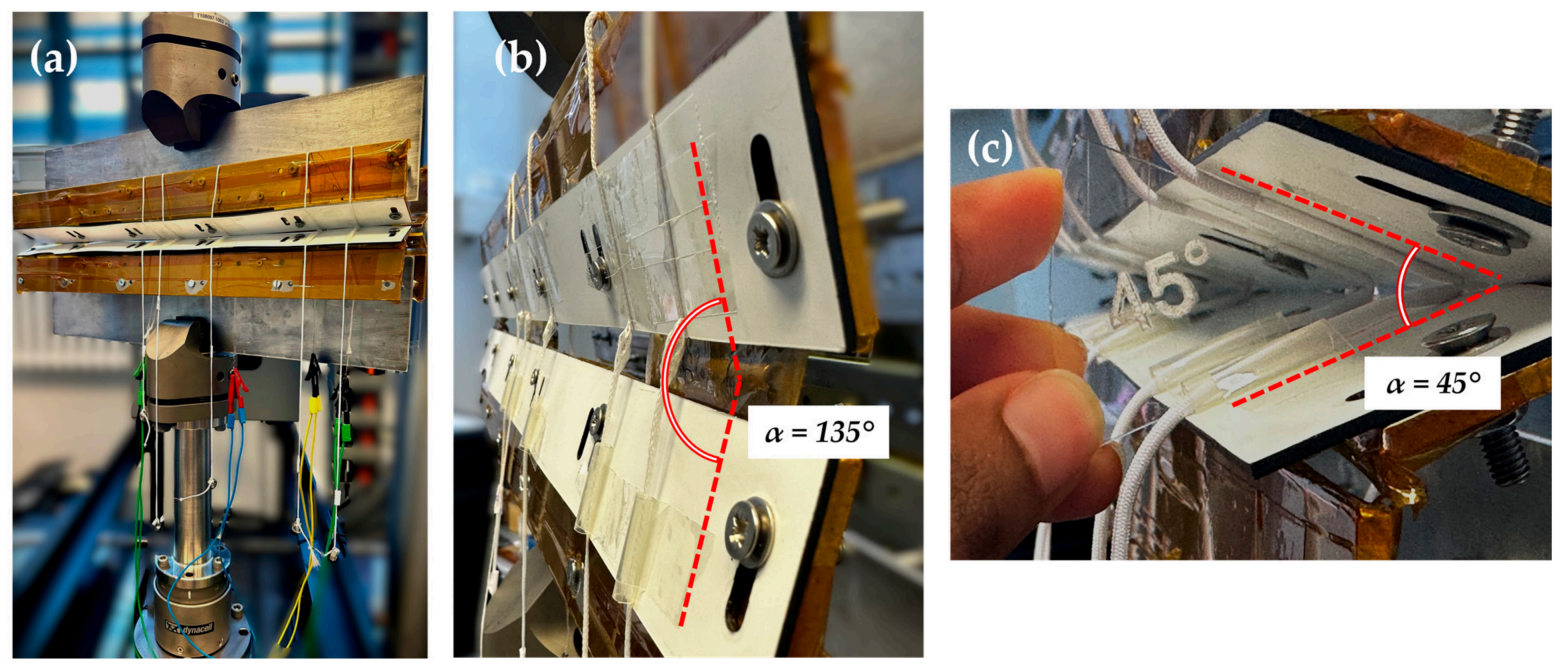
Experimental setup of the bending fatigue test. (**a**) Overall asymmetric mounting of the E-yarn between stationary and oscillating clamps with electrical connections for real-time functional monitoring; (**b**) E-yarn alignment in the recovery state; (**c**) E-yarn alignment in the bent state.

Samples using the MS configuration and the braided Litz samples were mounted using the same method. In these cases, both yarn ends were directly connected to the electrical measurement system using clips, and no additional mass was required, as the clips provided sufficient resistance to slippage. The complete experimental setup of the bending test is shown in Figure 5.

Cyclic bending was conducted at a frequency of 1 Hz (60 cpm) with five specimens from each E-yarn configuration to support comparison across different structural designs. For the ES LED yarns, ten specimens were tested to provide a larger dataset for assessing variability within this configuration. An additional set of ES LED yarns was also tested at a reduced frequency of 0.5 Hz (30 cpm) to investigate the influence of bending rate on mechanical response. As no universally adopted bending fatigue standard is currently established for electronic yarns, the 2500-cycle limit was selected as a comparative screening condition. This cycle limit was considered appropriate for the present yarn level assessment as the imposed loading condition combined a severe maximum angular bending deformation of 135° with a cycling frequency of 60 cpm.

Published E-textile studies incorporating bending assessments commonly use application-specific or custom-developed protocols, with cycle limits and failure criteria selected accordingly [37,39]. For selected configurations, testing was extended to 10,000 cycles to further examine fatigue behaviour beyond the initial screening limit. The ES RES configuration was extended based on the hypothesis that its denser braid structure may provide greater yarn confinement under cyclic bending, while the B Litz configuration was extended as a component-free reference to assess conductive wire behaviour without an embedded polymer-encapsulated component. Electrical functionality and structural integrity were monitored throughout testing to capture failure processes associated with solder joints, polymer encapsulation interfaces, and conductive wire transitions.

#### 2.2.2. Torsional Fatigue

**Figure 6:**
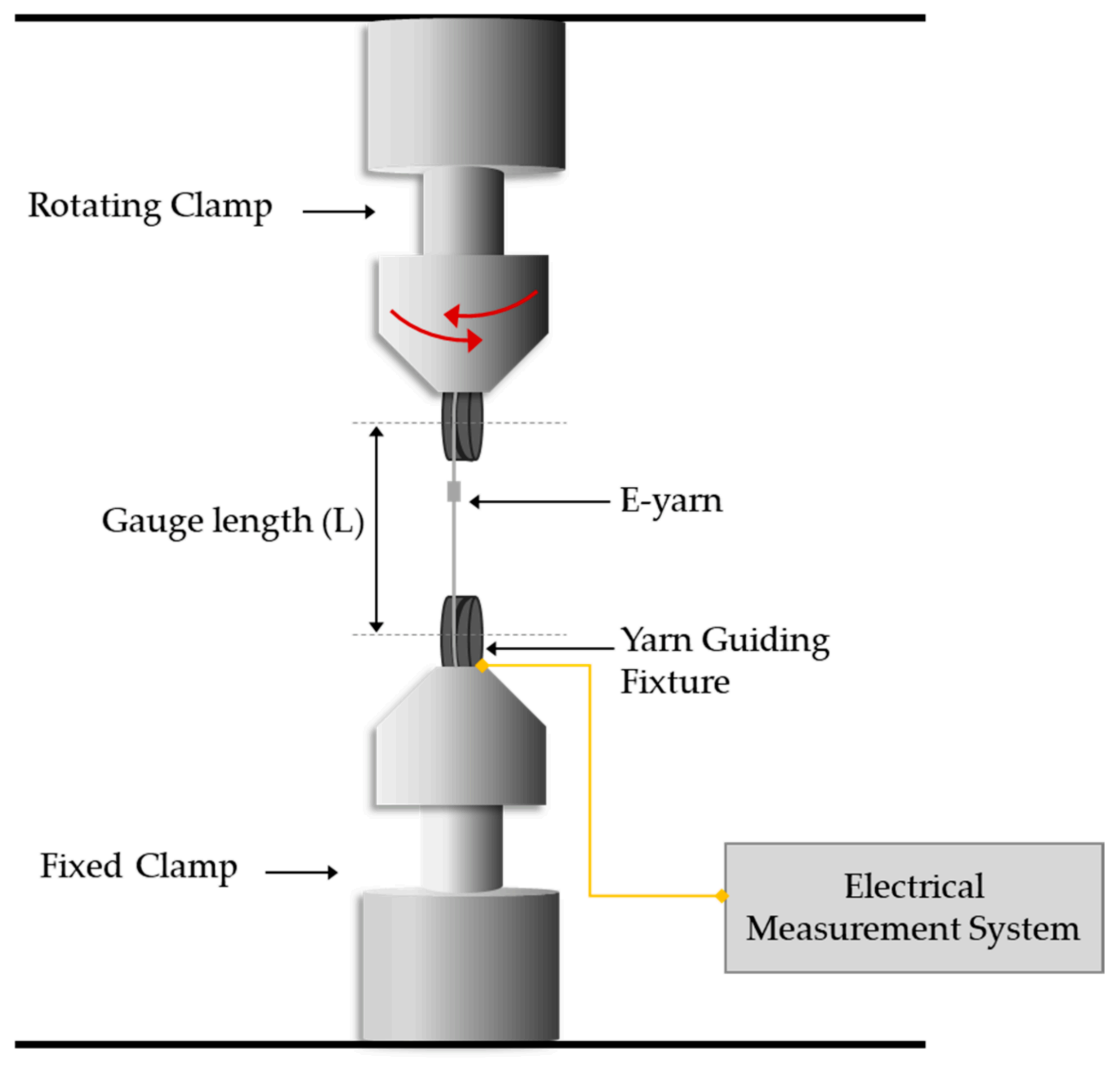
Schematic illustration of the torsional fatigue test configuration, showing the vertical mounting of the E-yarn between a stationary clamp and a rotational actuator, with arrows indicating its oscillating movement, enabling controlled cyclic twisting about the yarn’s longitudinal axis.

Torsional fatigue testing was designed to evaluate the durability of E-yarns when subjected to repeated twisting, a deformation mode that commonly arises from garment movement, drape, and rotation during wear. The same Instron ElectroPuls E10000 Linear-Torsion all-electric dynamic test system was used to apply controlled torsional loading. Five specimens from each E-yarn configuration were evaluated, excluding the MS LED configuration. Each yarn was oriented vertically within the testing system, with one end rigidly fixed in a stationary clamp, and the opposite end secured to the rotational actuator of the test system. This configuration enabled precise cyclic twisting about the longitudinal axis of the E-yarn, as illustrated in Figure 6.

For torsional loading, deformation severity is most appropriately captured using torsional shear strain, which directly reflects the extent of cyclic distortion imposed on the yarn structure. The shear strain at the yarn surface (*γ*) is given by

(4) $$\gamma =\frac{\theta \;r}{L},$$

where *θ* is the applied twist angle, *r* is the braided yarn radius, and *L* is the gauge length (as illustrated in Figure 6), which is the unsupported length of the E-yarn between the two clamping points subjected to torsional deformation.

To ensure that torsional deformation was concentrated at structurally critical locations, the influence of gauge length was systematically examined. Particular emphasis was placed on localising twist at the transition between encapsulated micro-pod regions and adjacent non-encapsulated yarn sections, which represent mechanically vulnerable interfaces. A gauge length of 35 mm was found to consistently focus torsional deformation within this region, whereas longer or shorter lengths distributed twisting more uniformly along the yarn, thereby reducing stress concentration at the intended failure site. This gauge length was therefore selected and applied across all tested E-yarn configurations.

**Figure 7:**
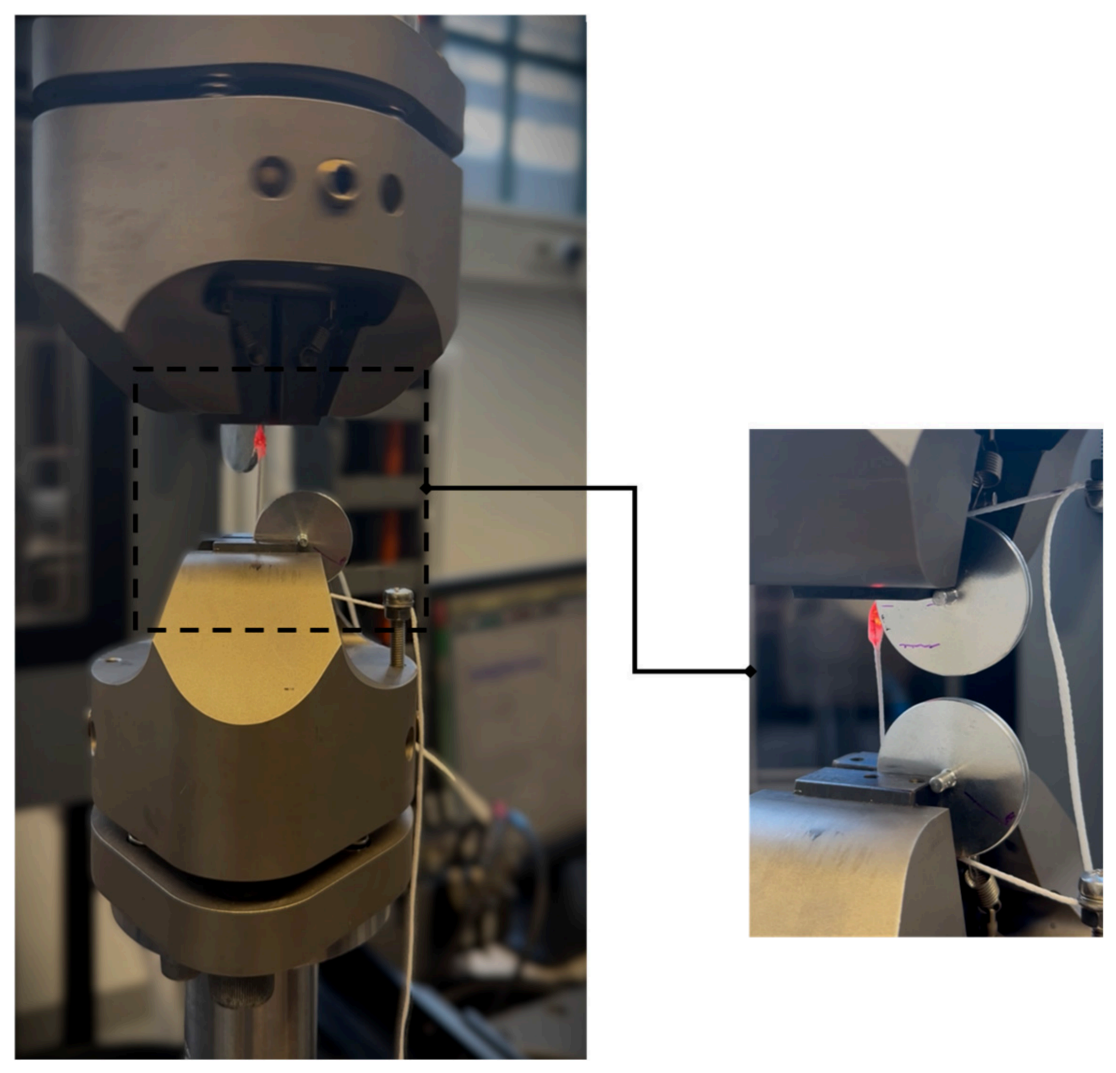
Experimental setup for the torsional fatigue test, showing the E-yarn secured between the fixed lower clamp and the rotational actuator, along with a close-up view of the E-yarn alignment.

Following the optimisation of the gauge length, torsional loading parameters were established. The maximum stable deformation attainable using the experimental setup corresponded to a bidirectional twist of ±250° at 0.5 Hz. This loading condition was adopted for all E-yarn configurations, as it imposed a demanding torsional cycle representative of extreme, yet plausible, textile-induced twisting. Although torsional fatigue protocols for E-textiles are not currently standardised, studies on fibre-based electronics have assessed functionality under a 180° twisting condition, investigated for 4000 twist cycles [40]. In the present study, the 2500-cycle limit was selected as an initial comparative torsional fatigue screening condition, consistent with the bending fatigue test limit, with selected configurations tested up to 10,000 cycles to examine their response under prolonged twisting based on the same hypothesis used in the bending fatigue test. The investigators considered this a reasonable number of twist cycles, as in a practical application, the E-yarn would be embedded securely within a textile structure and fibre–fibre friction between the textile and yarn would minimise torsional deformation. The actual torsional test setup is presented in Figure 7. Throughout testing, both electrical functionality and mechanical integrity were continuously monitored using the same failure criteria applied in the bending fatigue experiments (as detailed in Section 2.2.5).

#### 2.2.3. Quasi-Static Tensile Testing

Quasi-static uniaxial tensile testing was carried out to characterise the tensile response of the different E-yarn types, including strain at failure, force at failure, and the sequence of functional and mechanical failure. In contrast to the bending and torsional fatigue experiments, which focused on durability under repeated deformation, the tensile tests were designed to assess mechanical and functional failure under progressively increasing axial loading. This loading mode is representative of isolated high-strain events that may occur during wear, handling, donning or doffing, or stretching of wearable E-textiles.

**Figure 8:**
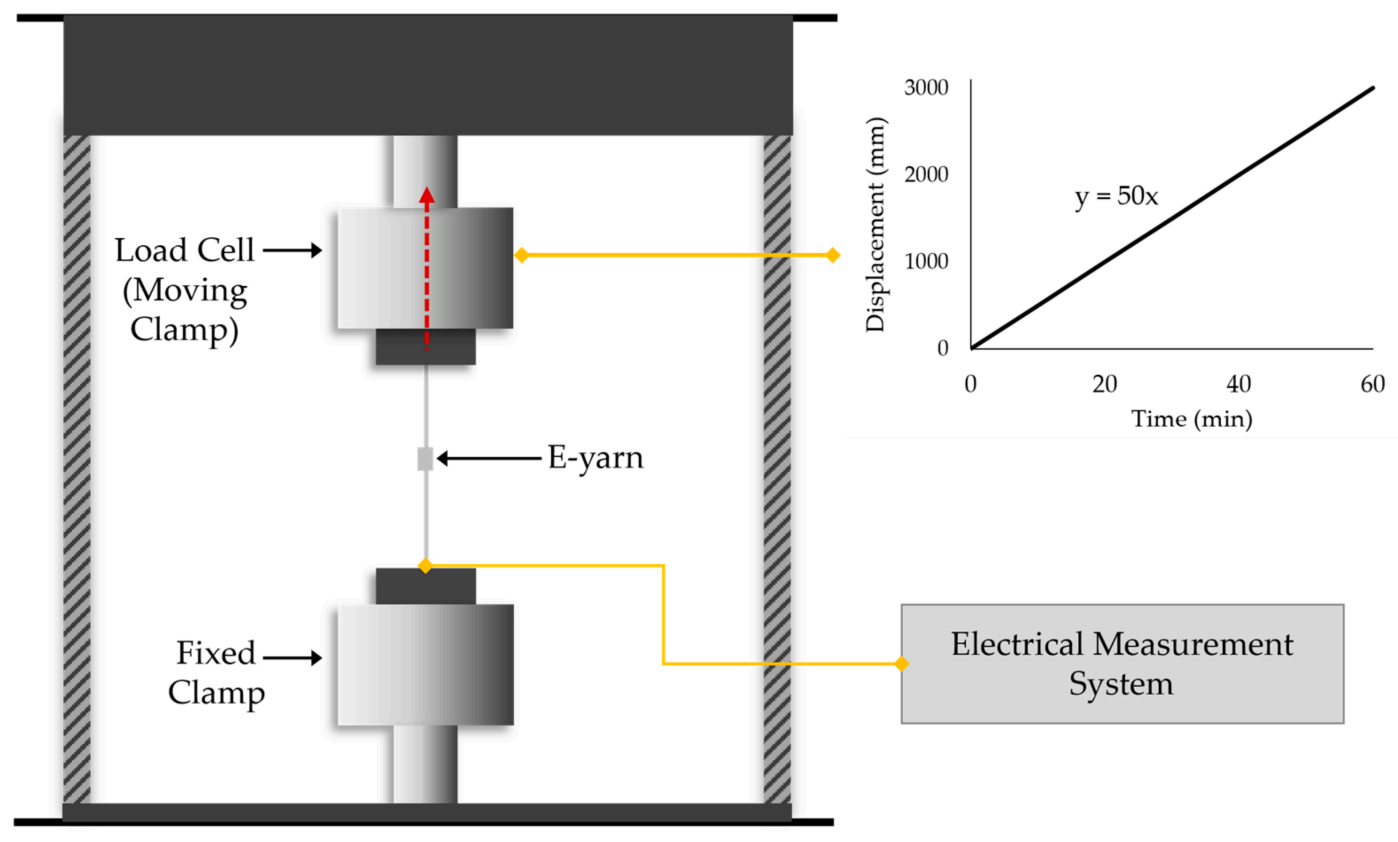
Schematic of the tensile test, illustrating the upward axial displacement applied from the load cell to the E-yarn along its length and the connection to the electrical measurement system.

**Figure 9:**
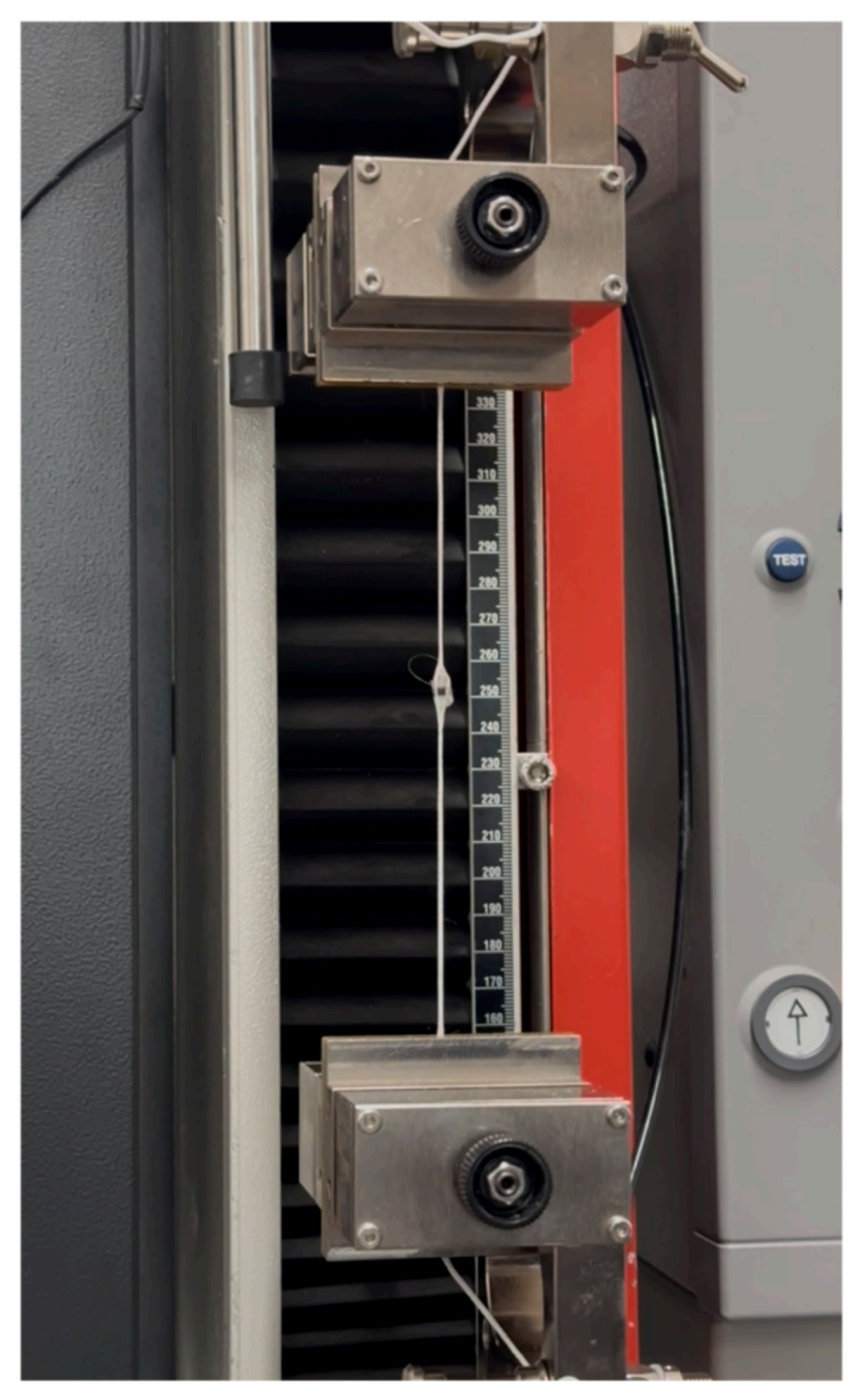
Experimental setup for the tensile test, showing the E-yarn secured between the grips of the testing machine for controlled axial loading.

The tensile experiments were performed using a ZwickRoell ZwickiLine Z2.5 TN+ universal testing machine (ZwickRoell GmbH & Co. KG, Ulm, Germany), following an adapted version of ISO 2062:2009 [41], which specifies procedures for determining the breaking force and elongation of textile yarns. This method has been used in previous E-yarn testing (for example [7]). A 200 N load cell was employed in the test setup, with a crosshead displacement rate of 50 mm min^−1^ and a gauge length of 150 mm. This corresponded to a nominal strain rate of 5.56 × 10^−3^ s^−1^. The selected displacement rate was chosen to maintain stable gripping of the braided E-yarn specimens and to minimise grip slippage and grip-related premature failure, which can occur during tensile testing of conductive and composite yarn structures. Similar adaptations to the tensile test standard conditions have been reported in electromechanical characterisation of conductive yarns [28,42]. Five specimens from each E-yarn configuration were tested to ensure reliable comparisons. The experimental setup is shown schematically in Figure 8, and the actual test setup is presented in Figure 9.

Throughout testing, both structural failure of the textile yarn assembly and loss of electrical functionality were monitored. This combined mechanical and functional evaluation enabled direct correlation between textile assembly rupture and electronic failure, allowing identification of whether functional performance degradation occurred prior to, or coincident with, mechanical breakage.

The tensile strain (*ε*) was calculated using the standard relationship:

(5) $$\varepsilon \;=\frac{\;\Delta L}{L_{0}},$$

where ∆*L* is the elongation at any given point, and *L*_0_ is the initial gauge length. Under quasi-static tensile loading, strain quantifies the relative extension of the yarn, providing a direct measure of deformation progression until rupture.

#### 2.2.4. Wash Durability

Wash durability testing was conducted to evaluate the functionality of E-yarns under combined mechanical, chemical and hydrothermal stresses encountered during domestic laundering. The washing and drying procedures were carried out in accordance with BS EN ISO 6330:2021 [43], which is widely used to assess the launderability of textile integrated electronic systems (for example [17]).

All samples were washed using a front-loading (horizontal axis Type A) household washing machine (Bosch Logixx 8, Robert Bosch UK Holdings Ltd., Uxbridge, UK) operating under standard domestic conditions. Each wash cycle consisted of a wash stage of 15 min at 40 °C, followed by a 10 min rinse stage and a 6 min spin cycle at 800 rpm, representing a realistic laundering scenario for wearable textiles. A total wash load of 2.01 kg was maintained for each cycle. The load consisted of five specimens from each component-embedded E-yarn configuration, with each specimen placed individually in a colour-coded protective wash bag according to its E-yarn configuration. Braided Litz samples were excluded because their wash response would not directly assess the durability of component-embedded E-yarn architectures. Ballast material made from 100% polyester plain woven fabric (290 GSM), cut into pieces of approximately (21 ± 4) cm × (21 ± 4) cm and overlocked on all edges, was added to achieve the required total load. A commercially available detergent (Persil Non-Bio Washing Powder, Unilever UK Ltd., Kingston Upon Thames, UK) was used at a fixed dosage of 20 g to ensure consistent chemical exposure across all tests.

Following each wash cycle, samples were air dried (Procedure A—Line dry) at ambient laboratory conditions (17 ± 2 °C, 62 ± 4% RH) within the wash bags to minimise handling-induced damage while the samples were wet. The wash durability tests were conducted over 25 consecutive wash–dry cycles, a cycle count selected based on its widespread use in the E-textile literature for evaluating laundering durability and functional retention. E-yarn functionality was assessed after each cycle. This methodology has also been adopted in previous studies involving E-yarn-based textiles.

#### 2.2.5. Functionality Verification

To assess the electrical functionality of E-yarns during mechanical durability testing, component-specific electrical parameters were identified and monitored throughout the experiments. The electrical monitoring strategy was selected to capture both gradual performance degradation and sudden electrical failure, employing a combination of continuous data acquisition and periodic measurements depending on the electrical characteristics of each E-yarn type.

For LED E-yarns, electrical resistance was monitored as the primary indicator of the conductive pathway integrity, while visual illumination of the LED was used to confirm functional operation. Illumination was verified by powering the LED using the diode test mode of the measurement system. Although resistance does not directly quantify luminous output, it provides a sensitive measure of damage accumulation within the conductive network. For MS LED E-yarns, where conductive wires extend from the embedded LED to both ends of the yarn, functionality was assessed at both ends. Since both conductive pathways are necessary for the complete E-yarn assembly to function as intended, loss of electrical continuity on either conductive path was classified as functional failure of the E-yarn. Progressive increases in resistance were interpreted as indicative of fatigue development at potential weak points, whereas abrupt discontinuities corresponded to loss of electrical continuity or complete failure.

In the case of PD E-yarns, functional performance was assessed by measuring the output voltage under constant illumination. Ambient overhead lighting was used as the light source, with fixed positioning of the test apparatus to ensure reproducible exposure. The recorded voltage response was used as a measure of PD responsivity, defined as the ratio of electrical output to incident optical power. A reduction in voltage output under identical illumination conditions over successive loading cycles was interpreted as degradation of the PD-based E-yarn functionality.

Resistor-based E-yarns, incorporating resistors of nominal resistance 10 kΩ, were employed as reference samples representative of a typical two-terminal E-yarn architecture. For these E-yarns, resistance was continuously monitored to assess electrical stability during mechanical loading.

For braided Litz samples, electrical functionality was assessed by measuring the resistance of the individual Litz wires. Each braided Litz specimen contained two conductive wires; therefore, although five yarn specimens were tested, ten individual conductive paths were monitored. Failure percentages for the braided Litz category were calculated based on the number of failed conductive paths rather than the number of yarn specimens.

The total electrical resistance of a general E-yarn can be expressed as

*R*_*total*_ = *R*_*Litz*_ + Σ*R*_*Solder*_ + Σ*R*_*Clip*_, (6)

where *R_Litz_* represents the resistance of the conductive Litz wires, *R_Solder_* corresponds to discrete resistances at solder joints, and *R_Clip_* denotes contact resistance at measurement interfaces. Given that *R_Total_* is typically on the order of 10^2^ Ω, whereas degradation at an individual solder joint occurs at the milliohm scale (Δ*R_Solder_* ≈ 10^−3^ Ω), such small changes are not readily resolvable through whole yarn resistance measurements. Nevertheless, sudden changes in resistance or voltage output provide a reliable indicator of final electrical failure.

The electrical measurement approach during the fatigue tests, whether automated or manual, was selected according to the resistance range of each E-yarn type. E-yarns exhibiting resistance values below 200 kΩ, including resistor-based E-yarns, were connected to a two-terminal custom-made resistance measurement system capable of multiplexing up to sixteen channels, with data recorded at 40 ms intervals throughout testing. This enabled continuous, real-time detection of both progressive degradation and abrupt electrical failure. For higher resistance E-yarns, electrical measurements were obtained manually at 100-cycle intervals using a digital multimeter (Fluke 179 True RMS Multimeter, Fluke Europe B.V., Eindhoven, The Netherlands). This measurement strategy ensured consistent data quality across all E-yarn types and facilitated reliable comparison between electrical degradation and mechanical fatigue behaviour.

To monitor E-yarn functionality during quasi-static tensile loading, electrical measurements were acquired using a Keithley DAQ6510 data acquisition system (Tektronix UK Ltd., Oldbury, UK) in combination with a Keithley 7710 solid-state differential multiplexer. Once electrical failure was detected, electrical data acquisition was set to automatically terminate, while mechanical loading continued until the textile yarn assembly ruptured. This approach enabled clear differentiation between functional failure of the embedded electronic components and structural failure of the E-yarn.

Functionality monitoring was also performed during wash durability testing. For each E-yarn type, functionality was checked and the electrical measurements were recorded prior to washing and after each wash cycle to quantify functional degradation induced by laundering. Measurements were conducted using the same data acquisition system employed for tensile testing, ensuring consistency across all experimental protocols. This approach enabled direct correlation between wash induced mechanical and environmental exposure and changes in E-yarn electrical performance over successive laundering cycles.

#### 2.2.6. Structural and Morphological Failure Characterisation

Structural and morphological characterisation was undertaken to support interpretation of the mechanical reliability and durability results by examining both the initial structural state of the E-yarns and the damage features associated with failure. To verify manufacturing quality and internal electrical integrity, X-ray imaging was performed on all E-yarn samples prior to mechanical reliability testing. These pre-test inspections focused specifically on evaluating the quality of solder joints confirming continuity between the embedded components and conductive Litz wires, ensuring that subsequent failures could be attributed to testing induced damage rather than fabrication related defects.

X-ray imaging of E-yarns subjected to bending and torsional fatigue testing was carried out using a Phoenix Xlaminer Industrial Microfocus X-ray Inspection System (Waygate Technologies, Baker Hughes Company, Houston, TX, USA) at Fraunhofer IZM, while X-ray imaging of samples used in tensile and wash durability testing was performed using a Nikon XT H 225 System (XT H Series-Nikon Metrology, Leuven, Belgium) at Nottingham Trent University. Comparable imaging conditions were maintained across both facilities to ensure consistent internal structural assessment.

Following durability testing, E-yarn samples that exhibited functional failure were subjected to post-test morphological analysis. Optical microscopy (VHX-5000; Keyence, Osaka, Japan) was first employed to inspect external damage features to the E-yarn. To further assess internal damage mechanisms, X-ray imaging was repeated on selected failed samples, enabling non-destructive visualisation of degradation such as solder joint fracture, conductor breakage, and displacement of embedded components.

For selected specimens, scanning electron microscopy (SEM) was subsequently used to investigate localised failure features and fracture surface morphology. SEM analysis was performed using a Phenom XL G2 Desktop SEM (Thermo Fisher Scientific Inc., Waltham, MA, USA) at Fraunhofer IZM for the bending and torsional fatigue test samples, and a JSM-7100F FEG SEM (JEOL Europe BV, Zaventem, Belgium) at Nottingham Trent University for the tensile and wash durability samples.

Together, these complementary techniques enabled assessment of the internal integrity of the E-yarns before testing, followed by detailed examination of structural damage and fracture features after testing. This supported comparison of failure mechanisms arising from mechanical fatigue, quasi-static tensile loading, and wash durability tests.

#### 2.2.7. Finite Element Analysis

A simplified finite element model (FEM) was developed using the Static Structural module in Ansys (2024 R2) to examine the bending behaviour of the polymer encapsulated E-yarn configurations. The model was used to better understand the mechanical response of the E-yarn during bending by identifying regions of maximum stress and strain accumulation in the conductive wire and polymer encapsulation. This was based on the hypothesis that functional failure under cyclic bending is primarily governed by the conductive Litz wires and the polymer-encapsulated component region, particularly at the transition between encapsulated and non-encapsulated sections.

Four models were developed to represent the main component embedded E-yarn configurations investigated experimentally. The encapsulation geometries were based on the corresponding pod dimensions used experimentally. To reduce computational complexity, the surrounding textile braid and Vectran supporting yarn were omitted. The Litz wire was also idealised as a single copper conductor rather than the actual twisted multistrand structure, thereby excluding strand-to-strand contact, enamel insulation, textile wrapping, braid-core interaction, and manufacturing variability.

The simulations were performed as a three-dimensional static structural analysis corresponding to the deformation from the recovery state to the bent state. The modelled geometry was assigned a non-linear copper alloy material. The mesh was generated using the automatic mechanical meshing method with adaptive sizing. The bending deformation was applied using fixed support and remote displacement boundary conditions. The loading was defined over five sequential load steps, with rotation about the X-axis progressively increased to 135°, representing the maximum bent state used in the experimental bending fatigue test. Rotations about the Y and Z axes were constrained to 0° during the imposed deformation. Equivalent stress, equivalent strain and total deformation were extracted from the solution to evaluate localised mechanical loading within each E-yarn configuration. The FEM was therefore used to examine local stress and strain distributions within the simplified E-yarn geometry, rather than to provide a quantitative prediction of fatigue life.

## 3. Results and Discussion

### 3.1. Bending Fatigue Behaviour

**Figure 10:**
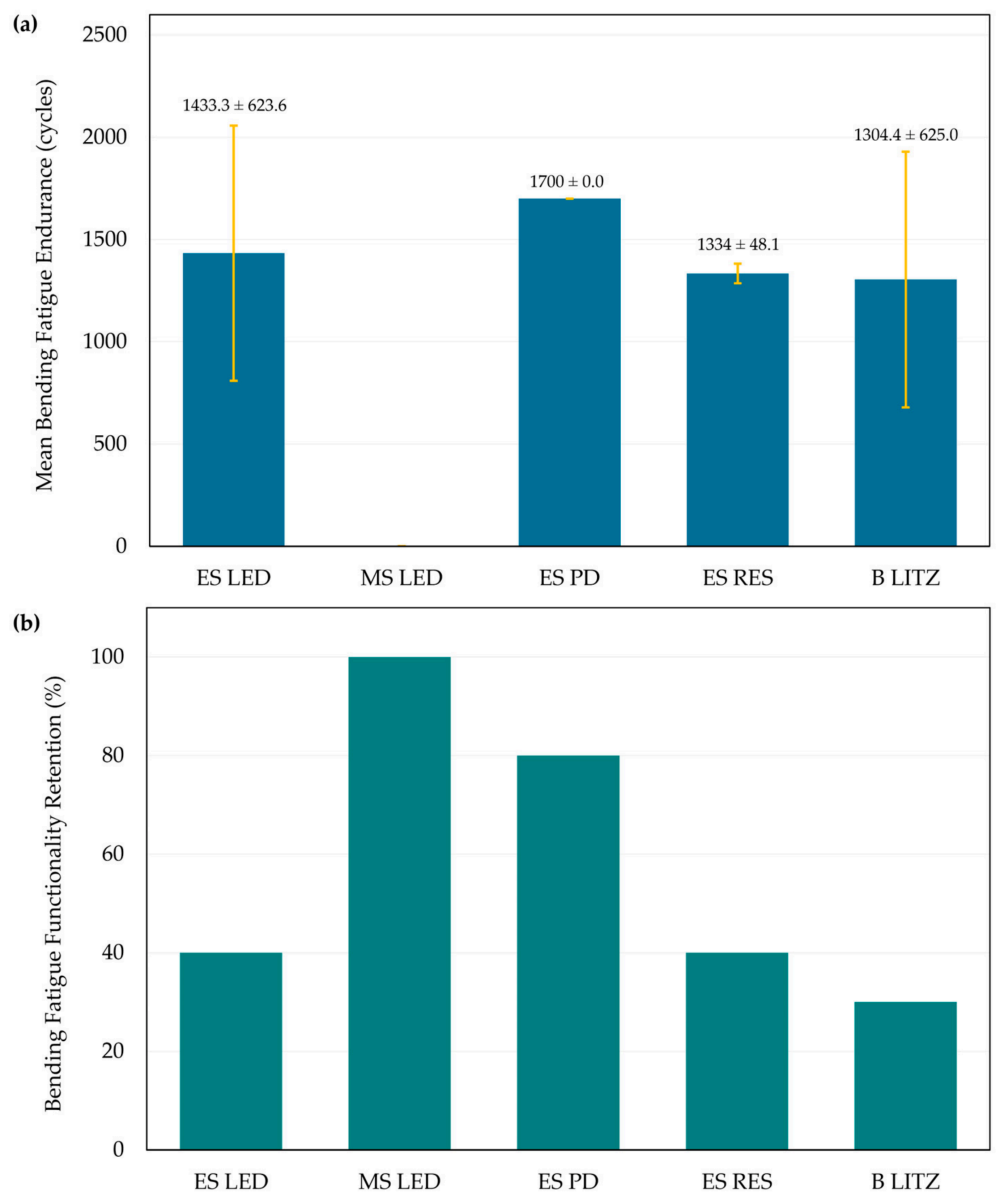
Bending fatigue performance of E-yarn configurations. (**a**) Mean fatigue endurance within the 2500-cycle test limit, with standard deviation indicated by error bars; (**b**) percentage of samples retaining functionality at the 2500-cycle limit.

The bending fatigue performance of the E-yarn categories is summarised in Table 2, which reports the percentage of failed samples at the 2500-cycle limit and the corresponding average number of cycles to failure under identical bending conditions. In addition, extended testing of the braided Litz and resistor E-yarns up to 10,000 cycles is included to provide further insight into the fatigue behaviour of the Litz conductors and the possible contribution of the braided textile structure to strain redistribution. The bending performance of each E-yarn configuration is presented in Appendix A. The mean bending fatigue failure endurance and functionality retention of the E-yarn configurations are shown in Figure 10a and Figure 10b, respectively.

For a more meaningful comparison, the bending fatigue behaviour of all E-yarn categories was analysed within the 2500-cycle limit (providing a common comparative boundary across the tested configurations, while the extended data collected up to 10,000 cycles are presented for completeness). Clear differences in bending fatigue resistance were observed between the E-yarn configurations. MS LED E-yarns exhibited the highest resistance to bending fatigue, with no failures recorded within the test limit. In contrast, ES LED and ES resistor E-yarns showed a markedly higher failure incidence, with 60% of samples failing within the same test limit and an average failure occurring at approximately 1383 cycles under the same bending conditions, despite having different braid covering configurations. Braided Litz wires with no embedded components failed within a similar cycle range, with a failure rate of 70%. ES PD E-yarns showed comparatively higher functionality retention, with only 20% of samples failing within the 2500-cycle limit.

The improved bending response of the MS LED configuration may be attributed to its more balanced wire arrangement as shown in Figure 2. In the ES LED configuration, the Litz wires terminate at the component, creating a localised transition at the solder joint and encapsulation boundary where bending strain could concentrate. In contrast, the MS LED configuration has continuous Litz wires extending on both sides of the component, which may facilitate a more uniform distribution of deformation along the yarn structure during bending. The absence of MS LED failures over 2500 bending cycles is consistent with this interpretation; however, the underlying stress redistribution mechanism cannot be conclusively established from the experimental observations alone. Furthermore, the simplified FEM analysis discussed later in this section did not provide direct confirmation of this mechanism.

ES LED and ES PD E-yarns shared identical braid constructions, conductive wire architectures, and encapsulation materials but differed in polymer encapsulation pod geometry. These configurations exhibited different bending fatigue responses, with average failures occurring between approximately 1400 and 1700 cycles. A difference in failure incidence was observed, with 60% of ES LED E-yarns failing compared with 20% of ES PD E-yarns within the same bending test limit. Since the braid construction, conductive architecture, encapsulation material, and testing conditions were held constant between these two configurations, this difference suggests that the encapsulation pod geometry may have influenced the probability of failure under cyclic bending. However, this interpretation should be treated as a design-related observation rather than a statistically confirmed independent effect.

Finite element modelling was used to examine the local mechanical response of the simplified E-yarn geometries during bending. The equivalent stress and strain distributions showed that the highest values developed along the conductive wire path within the imposed bending region, which was positioned close to the polymer encapsulation exit. Therefore, the FEM results describe stress and strain distribution under the selected bending configuration, rather than independently identifying the encapsulation boundary as the only critical failure region. However, the location of higher stress and strain was consistent with the post-failure X-ray observations, where fractures were mainly observed in the braided Litz wire region near the encapsulation transition. A video illustrating the FEM simulation of bending deformation is provided as Supplementary Video S1.

**Figure 11:**
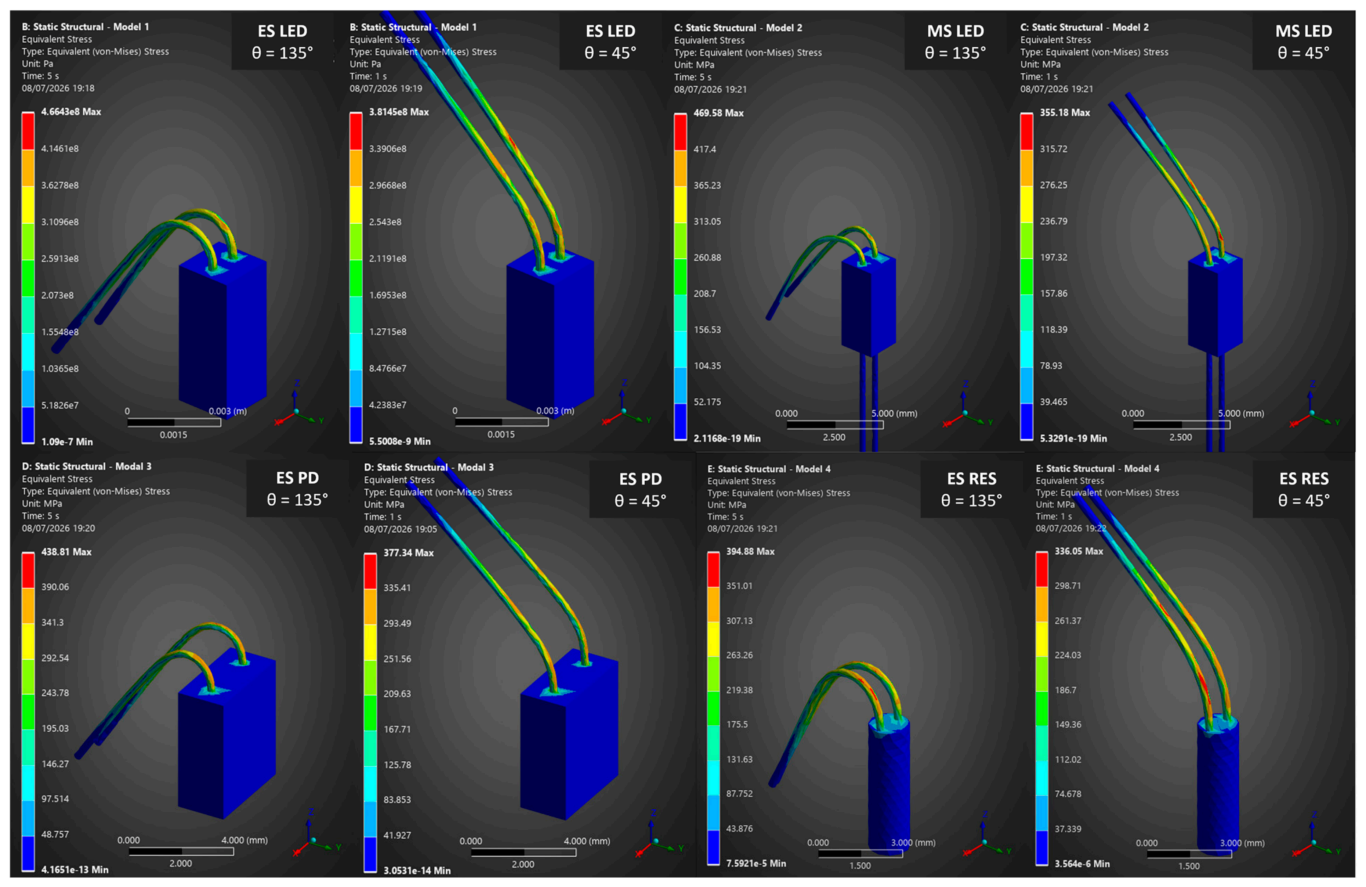
Finite element contour plots of equivalent stress distribution during bending, with results shown at θ = 135° (bent state) and θ = 45° (recovery state) for each model. Images are reproduced directly from the FEM simulation output, with only figure labels added for clarity.

**Figure 12:**
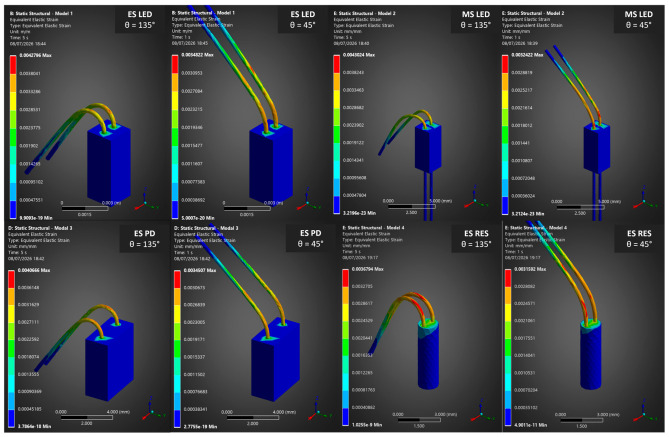
Finite element contour plots of equivalent strain distribution during bending, with results shown at θ = 135° (bent state) and θ = 45° (recovery state) for each model. Images are reproduced directly from the FEM simulation output, with only figure labels added for clarity.

The maximum equivalent stress and strain values increased from the recovery state to the bent state for all configurations as shown in Figure 11 and Figure 12 and summarised in Table 3, confirming that the bent state represented the most mechanically severe position within the simulated loading sequence.

The FEM-derived maximum stress and strain values did not directly match the experimental bending fatigue performance. For example, the MS LED model showed one of the highest peak stress and strain values, whereas the MS LED samples retained functionality throughout the 2500-cycle bending test. This indicates that peak stress or strain in this simplified model alone is not sufficient to explain the experimentally observed fatigue behaviour.

The differences between the FEM outcomes and the experimental fatigue behaviour are likely due to several factors that were not captured in the simplified model. The actual Litz wire consists of twisted multistrand copper conductors with enamel insulation and textile wrapping, whereas the model idealised the conductor as a single copper body. In addition, braid confinement, Vectran support, solder-joint geometry, wire–polymer interaction, strand-to-strand contact, and fatigue damage accumulation were not included. Manual fabrication and handling steps, such as soldering, encapsulation, respooling, and rewinding of the Litz wires, may introduce local geometric variation, tension variation, or pre-existing damage. These factors could also influence strain redistribution and fatigue failure in the experimental samples.

Overall, the FEM results should be considered a simplified numerical assessment of stress and strain distribution under the selected bending configuration. The model provides useful insight into the concentration of mechanical loading near the imposed bending region, close to the conductor exit from the encapsulation pod. However, it does not quantitatively predict the experimental fatigue ranking. More detailed modelling, including the multistrand Litz wire structure, braid confinement, polymer–wire interaction, solder geometry, and fatigue damage accumulation, would be required to improve the predictive capability of the model. Manufacturing variability must also be accounted for when assessing the fatigue ranking.

**Figure 13:**
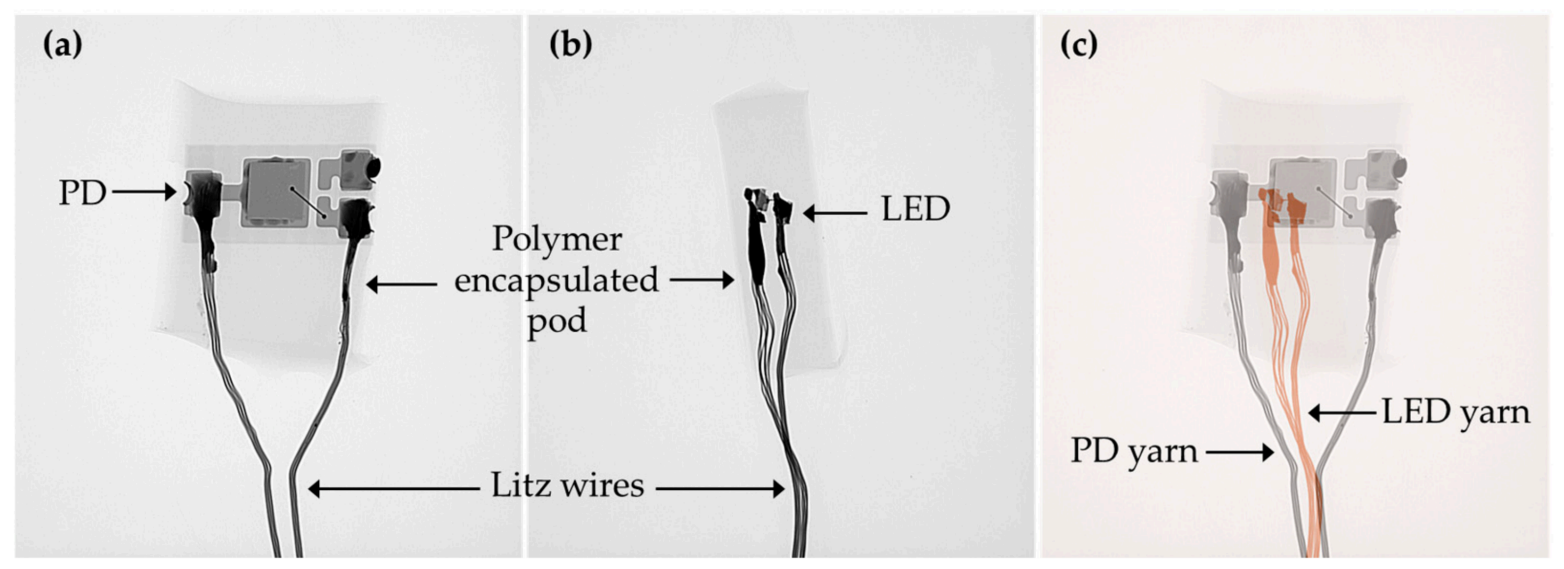
X-ray images showing differences in the transition profile of conductive Litz wires at the polymer encapsulation boundary in end-soldered E-yarns. (**a**) ES PD configuration; (**b**) ES LED configuration; (**c**) overlaid X-ray images illustrating differences in the Litz wire transition profile.

The non-destructive X-ray images in Figure 13 show that differences in encapsulation geometry affect the transition profile of the conductive Litz wires from the encapsulated region into the flexible yarn structure. In the narrower encapsulation used for LED E-yarns, the wires emerge over a shorter distance, whereas the wider encapsulation employed in PD E-yarns provides a more extended geometric transition between the encapsulated and non-encapsulated regions. Such geometric differences may influence the redistribution of bending-induced deformation at the encapsulation boundary, thereby affecting the likelihood of failure accumulation over repeated loading cycles.

**Figure 14:**
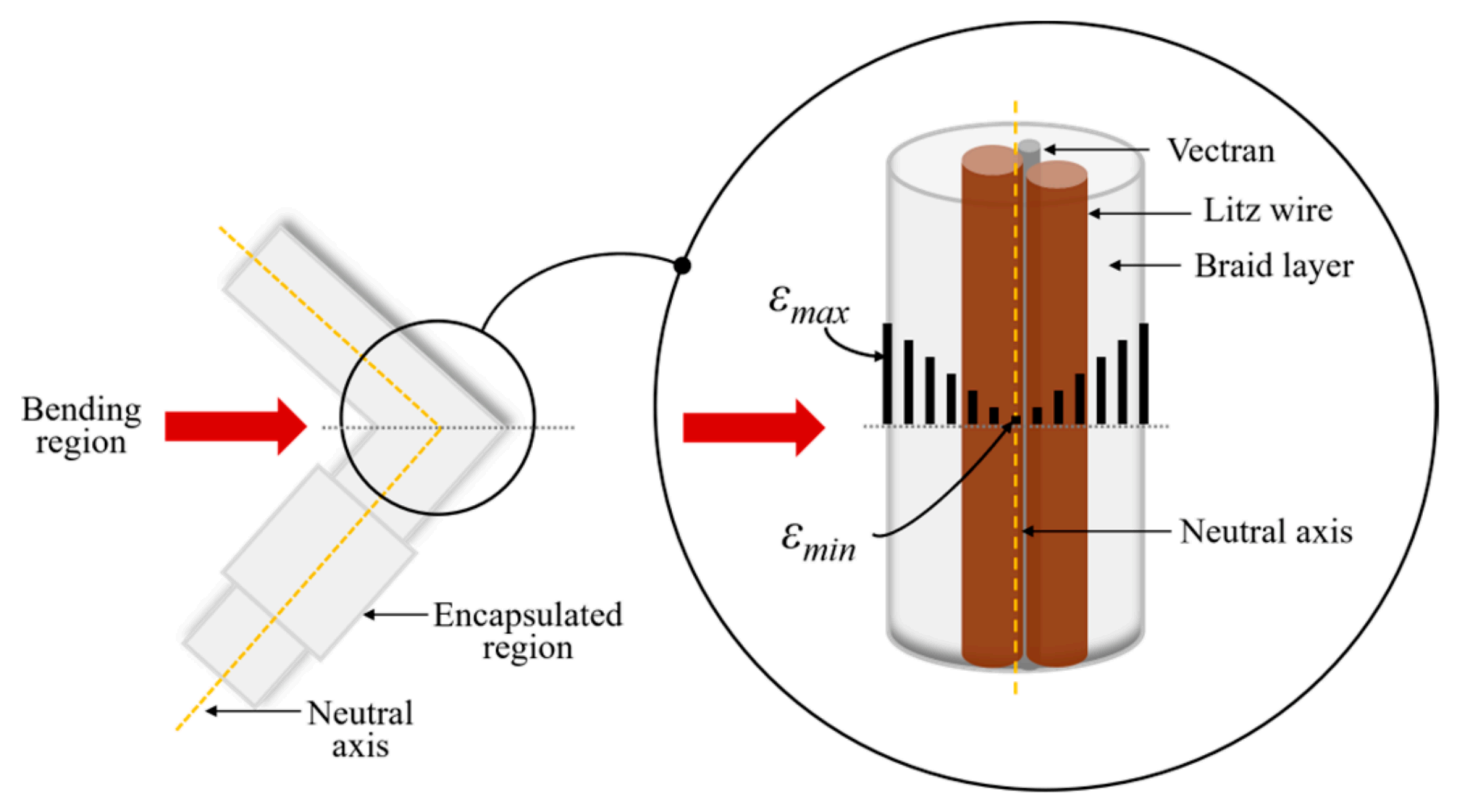
Schematic representation of the bending strain distribution across the cross-section of a general E-yarn in the bending region.

These observations can be interpreted in relation to the E-yarn internal architecture and strain distribution under bending deformation. In the non-encapsulated regions, the E-yarn core consists of two Litz wires and one Vectran yarn, which lie freely and in parallel within the surrounding textile braid. This forms a heterogeneous and non-consolidated structure in which uniform strain transfer across the cross-section cannot be assumed. As illustrated schematically in Figure 14, bending deformation is therefore expected to be distributed non-uniformly across the braid and internal conductive elements. However, comparison of the ES LED and ES RES E-yarns within the 2500-cycle limit does not demonstrate a consistent improvement in bending fatigue resistance with increased braid thickness, despite the thicker braid covering used in the ES RES configuration.

This non-uniform deformation is likely to be perturbed at material and geometric discontinuities, particularly at the boundaries between the polymer encapsulation and the non-encapsulated yarn regions. At these locations, changes in stiffness and geometry arise from the polymer micro-pod, embedded component, conductive wires, and surrounding braided textile structure, which may promote localised deformation and stress concentration. The observed differences in failure incidence between ES LED and ES PD E-yarns therefore suggest that encapsulation geometry may contribute to strain redistribution at these critical regions, potentially influencing fatigue under cyclic bending.

Statistical analysis of cycles to failure was conducted to evaluate the significance of differences in bending fatigue behaviour among E-yarn categories. The analysis was restricted to specimens that failed within the 2500-cycle test limit, as non-failed specimens represent right-censored observations. This approach enabled comparison of fatigue life among samples that progressed to failure within the defined test window. Pairwise comparisons were conducted using independent two-sample *t*-tests with unequal variances, selected to account for differences in sample size and variance between groups. A significance level of α = 0.05 was adopted, and the resulting *p*-values are presented in Table 4. Yarn configurations with no failure events (MS LED) were excluded from the comparison due to insufficient variability within the E-yarn configuration.

The pairwise comparisons of the E-yarn categories yielded *p*-values greater than 0.05, indicating that differences in mean cycles to failure were not statistically significant relative to within-group variability. Although variations in bending fatigue behaviour were observed across E-yarn categories, the current dataset does not provide sufficient statistical evidence to attribute these differences to specific factors such as encapsulation geometry, braid architecture, or component integration when the analysis is restricted to failed specimens. This analysis therefore evaluates cycles to failure among failed specimens and does not directly test failure incidence.

**Figure 15:**
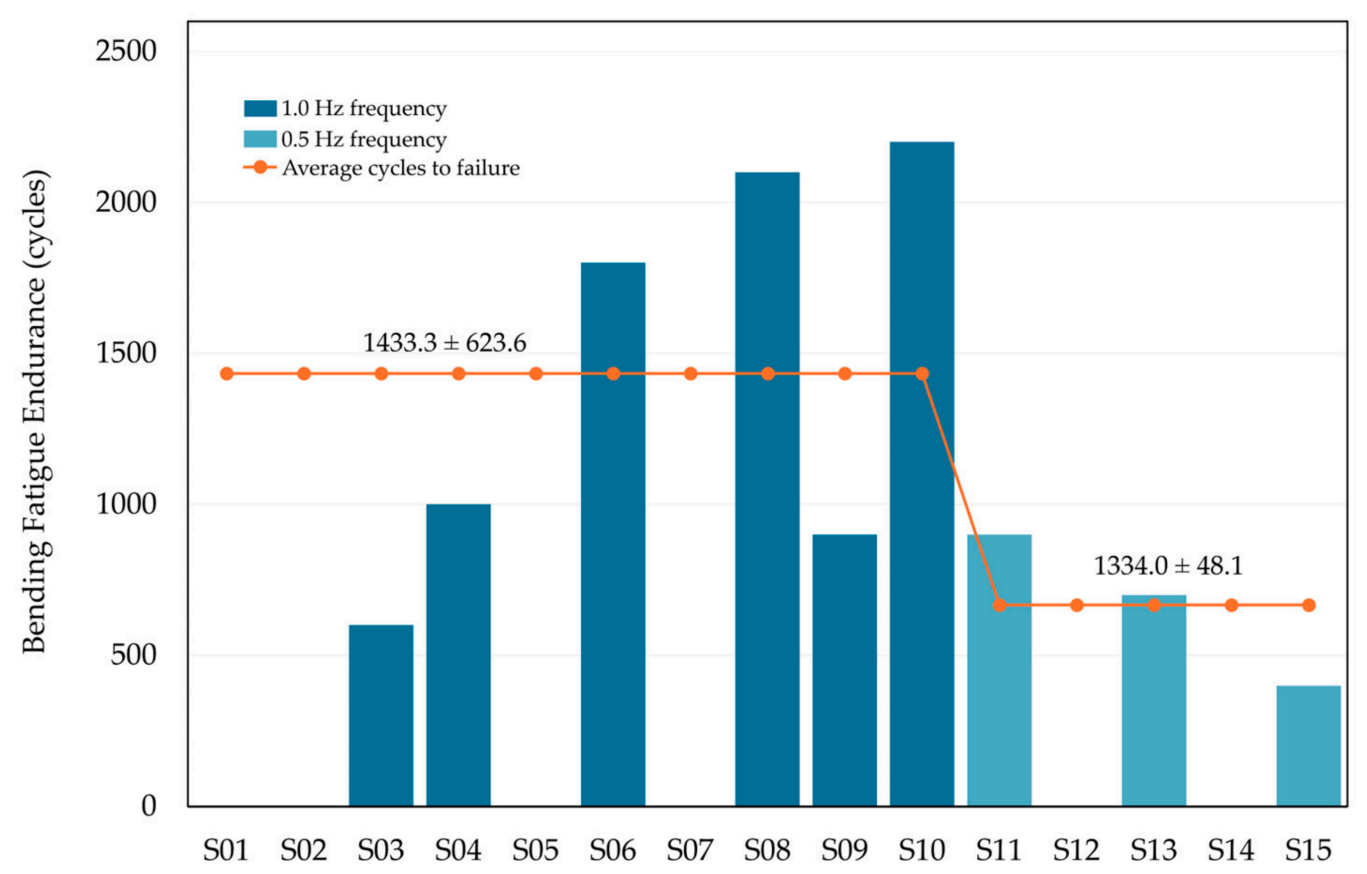
Cycles to failure for end-soldered LED E-yarn samples under cyclic bending at 1 Hz (S01–S10) and 0.5 Hz (S11–S15). Samples remaining functional at the test limit are shown as right-censored data. Average cycles to failure are presented with the scatter plot (the connecting line is for visual comparison and does not indicate a trend).

The influence of bending frequency on fatigue behaviour was assessed using ES LED E-yarns tested at both 1 Hz and 0.5 Hz, and the outcome is shown in Figure 15. Samples tested at the lower frequency failed at fewer cycles on average. This indicates that bending frequency influenced the fatigue response of the ES LED configuration within the tested conditions. One possible explanation is that the longer cycle duration at 0.5 Hz may allow greater time-dependent deformation or damage accumulation at the polymer encapsulation–conductor transition region.

This effect was further assessed statistically using a *t*-test. The observed difference in fatigue life between the two frequencies was found to be statistically significant (*p* < 0.05; given in Table 4), indicating that bending frequency exerts a measurable influence on fatigue performance within the analysed dataset.

**Figure 16:**
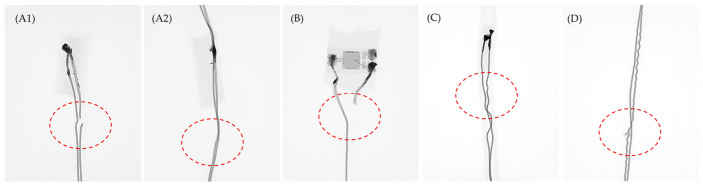
X-ray images of E-yarns after cyclic bending failure, with typical failure locations circled in red. (**A1**) End-soldered LED E-yarn. (**A2**) Mid-soldered LED E-yarn. (**B**) End-soldered PD E-yarn. (**C**) End-soldered resistor E-yarn. (**D**) Braided Litz wires.

Post-failure examination of the E-yarns subjected to bending fatigue revealed a consistent failure pattern across all yarn categories. X-ray images in Figure 16 indicated that fracture predominantly occurred within the braided Litz wire region at the boundary of the encapsulated segment, irrespective of yarn configuration. This localisation suggests that the transition between the encapsulated and non-encapsulated regions represents a mechanically sensitive zone under cyclic bending.

**Figure 17:**
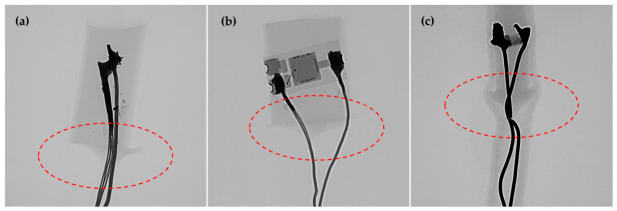
X-ray image analysis of encapsulation pod geometry, showing variations in polymer distribution and conductor alignment at the encapsulation exit region, including a conical feature (circled). Image contrast has been enhanced to distinguish the polymer encapsulation region. (**a**) End-soldered LED E-yarn. (**b**) End-soldered PD E-yarn. (**c**) End-soldered resistor E-yarn.

Pre- and post-failure X-ray image analysis revealed geometrical variations in the encapsulation micro-pod at the transition region between the polymer encapsulation and the flexible yarn section. The pod termination morphology was classified as straight, conical, or not clearly visible when the pod boundary could not be reliably identified from the X-ray images (Table A1). The conical morphology is considered a manufacturing-related geometrical variation at the encapsulation interface, likely associated with local polymer flow and curing behaviour during fabrication. This variation can alter the conductor exit profile by extending polymer coverage along the Litz wires beyond the primary encapsulation region, as shown in Figure 17.

**Figure A7:**
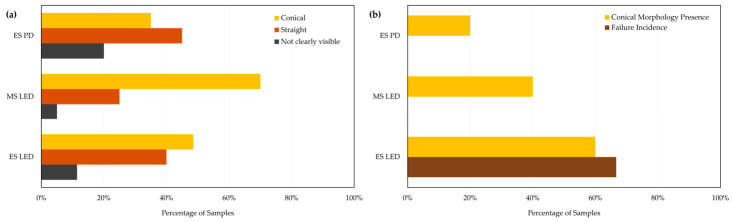
Distribution of encapsulation micro-pod termination morphologies and their occurrence in E-yarns tested for bending fatigue. (**a**) Pod termination morphology distribution of E-yarn configurations identified from X-ray image analysis; (**b**) conical morphology occurrence and corresponding failure incidence within the bending fatigue test subset.

This pod termination morphology classification was applied to the ES LED, ES PD, and MS LED samples fabricated and tested in this study, and the percentage incidence of each morphology is presented in Figure A7a. The ES RES samples were not included in this morphology quantification as their denser braid structure reduced X-ray visibility of the polymer pod boundary, making reliable classification difficult. Conical pod termination was observed in approximately 49% of ES LED samples, 70% of MS LED samples, and 35% of ES PD samples, indicating that this morphology occurred repeatedly rather than as an isolated observation. Within the bending fatigue sample subset, conical pod termination was present in 60% of ES LED samples, 40% of MS LED samples, and 20% of ES PD samples, as summarised in Table A2 and shown in Figure A7b. Among the conical samples, bending failure occurred in 66.7% of the ES LED samples, while no failures were observed in the MS LED or ES PD configurations within the 2500-cycle bending test limit. This indicates that conical pod morphology alone did not consistently correspond to premature failure across all E-yarn configurations. Due to the limited number of samples within each morphology group, these observations were used only to identify possible morphology-related failure trends and were not treated as statistically conclusive. Nevertheless, the observed geometrical variations may influence local strain distribution during deformation by altering stress transfer pathways and introducing localised stress concentrations, which could potentially contribute to differences in fatigue behaviour.

**Figure 18:**
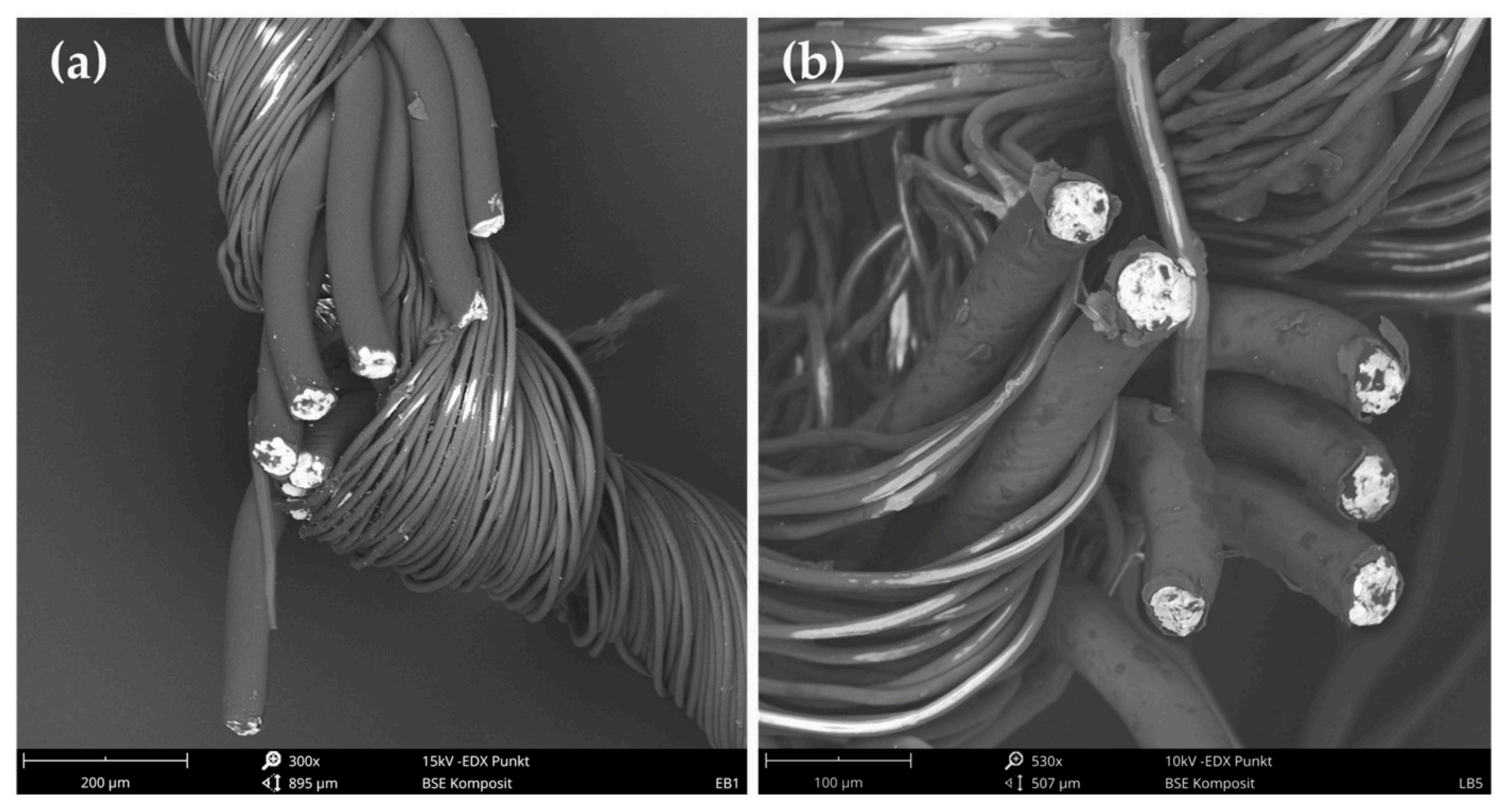
SEM images of fractured E-yarns following cyclic bending at increasing magnifications. (**a**) Fractured braided Litz wire region showing textile cover breakage, strand separation and fractured Cu strand ends; (**b**) higher magnification view of the fractured Cu strand surfaces, showing relatively flat fracture morphology without visible necking, indicating brittle fracture behaviour.

Further insight was obtained from SEM based surface morphology analysis of the fractured Litz wire regions as presented in Figure 18. The failure surfaces exhibited irregular and granular features with sharp facets, and no visible evidence of necking or plastic deformation. The relatively flat fracture profiles and the absence of elongation near the fracture edges are indicative of brittle fracture behaviour, characterised by rapid crack propagation with minimal plastic deformation. These features suggest that, under cyclic bending, the Cu strands within the Litz wires experience fatigue-induced damage accumulation, leading to embrittlement and abrupt fracture.

The electrical degradation observed during cyclic loading is likely to result from the combined effects of several mechanisms, including progressive damage to the Litz wire strands, conductor micro-cracking, solder joint degradation, and changes in contact resistance at measurement interfaces. However, as the measured response represents the whole E-yarn electrical pathway, individual contributions from solder fatigue, conductor damage, and contact resistance could not be fully separated. Therefore, electrical measurements were interpreted together with X-ray and SEM observations, which indicated that final failure under cyclic bending was mainly associated with Litz wire fracture near the encapsulation transition region.

### 3.2. Torsional Fatigue Behaviour

**Figure 19:**
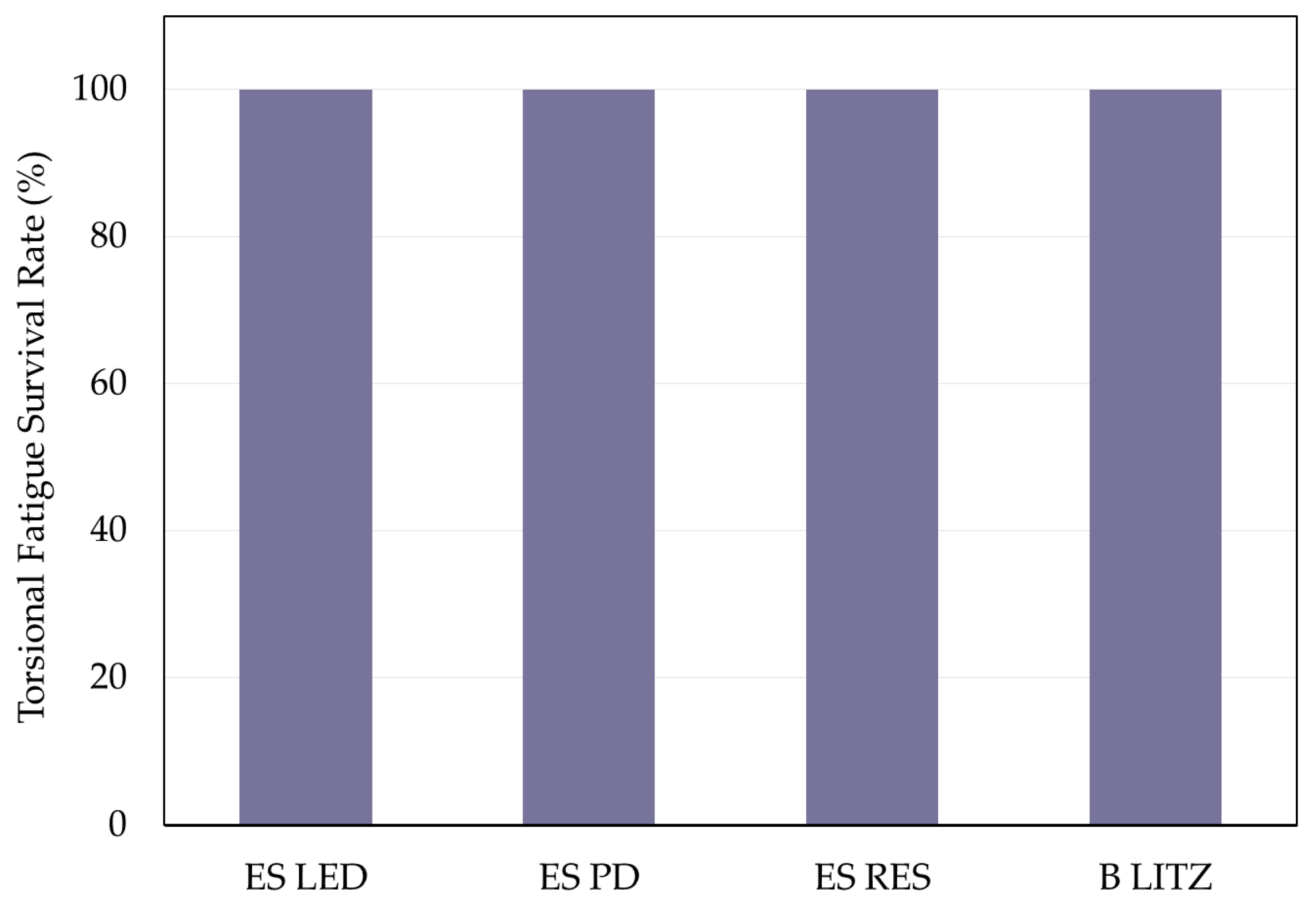
Torsional fatigue performance of E-yarn categories, showing percentage functionality retained within the specified 2500-cycle limit.

Under cyclic torsional loading, all tested E-yarn categories exhibited stable functional performance with no observable electrical or mechanical failures within the test limits as given in Table 5 and Figure 19. Specifically, samples tested up to 2500 cycles exhibited no failure at the defined test limit, while those evaluated over extended durations maintained functional integrity throughout the full 10,000 cycles.

The absence of torsional fatigue failure within the investigated test limits suggests that the tested E-yarn configurations were less sensitive to torsional loading than to cyclic bending under the conditions examined. During torsional loading, deformation may be distributed more gradually along the yarn axis and partly accommodated by relative movement between the internal yarn constituents and the surrounding textile braid. This may reduce the development of localised strain concentrations at the polymer encapsulation boundaries and solder joints compared with bending. Therefore, within the investigated loading regime, bending appeared to be the more critical cyclic deformation mode for functional failure in these polymer-encapsulated E-yarns.

**Figure 20:**
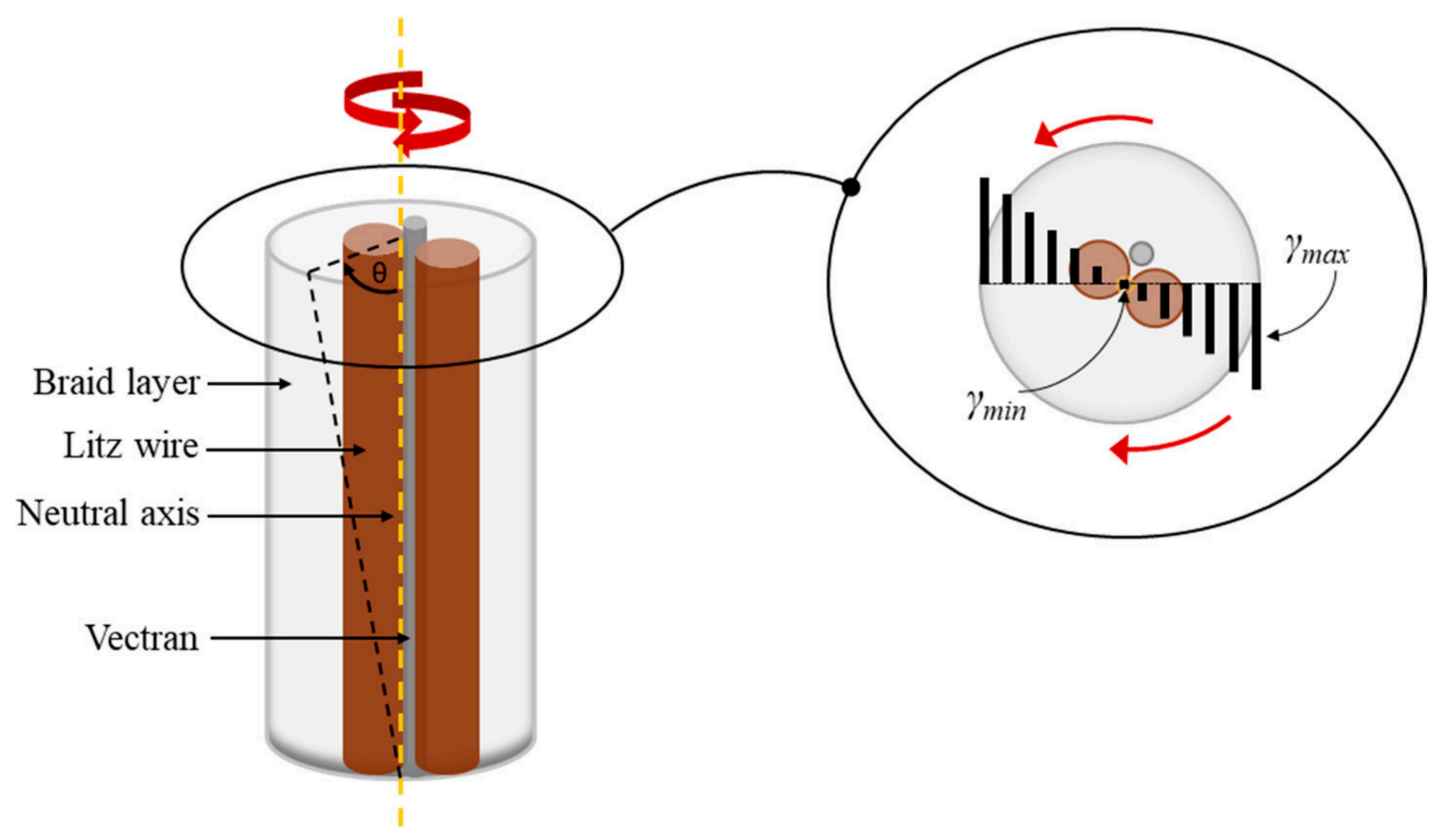
Schematic representation of torsional shear strain distribution across the cross-section of an E-yarn, illustrating the radial variation in shear strain and its influence on the deformation of internal components within the heterogeneous yarn structure. The red arrows indicate the bi-directional torsional torque.

From a mechanical perspective, torsional shear strain provides a more appropriate descriptor of the applied loading conditions than torsional stress, particularly for E-yarns, which exhibit a heterogeneous, non-consolidated composite architecture. As illustrated in Figure 20, torsional shear strain varies radially across the yarn structure and governs the deformation experienced by the individual constituents. In contrast, estimation of torsional stress requires a defined shear modulus, which is not well established for multi-component systems such as E-yarns. Consequently, strain-based metrics provide a more robust and geometry-dependent basis for evaluating fatigue behaviour under torsional loading.

### 3.3. Quasi-Static Tensile Behaviour

**Figure 21:**
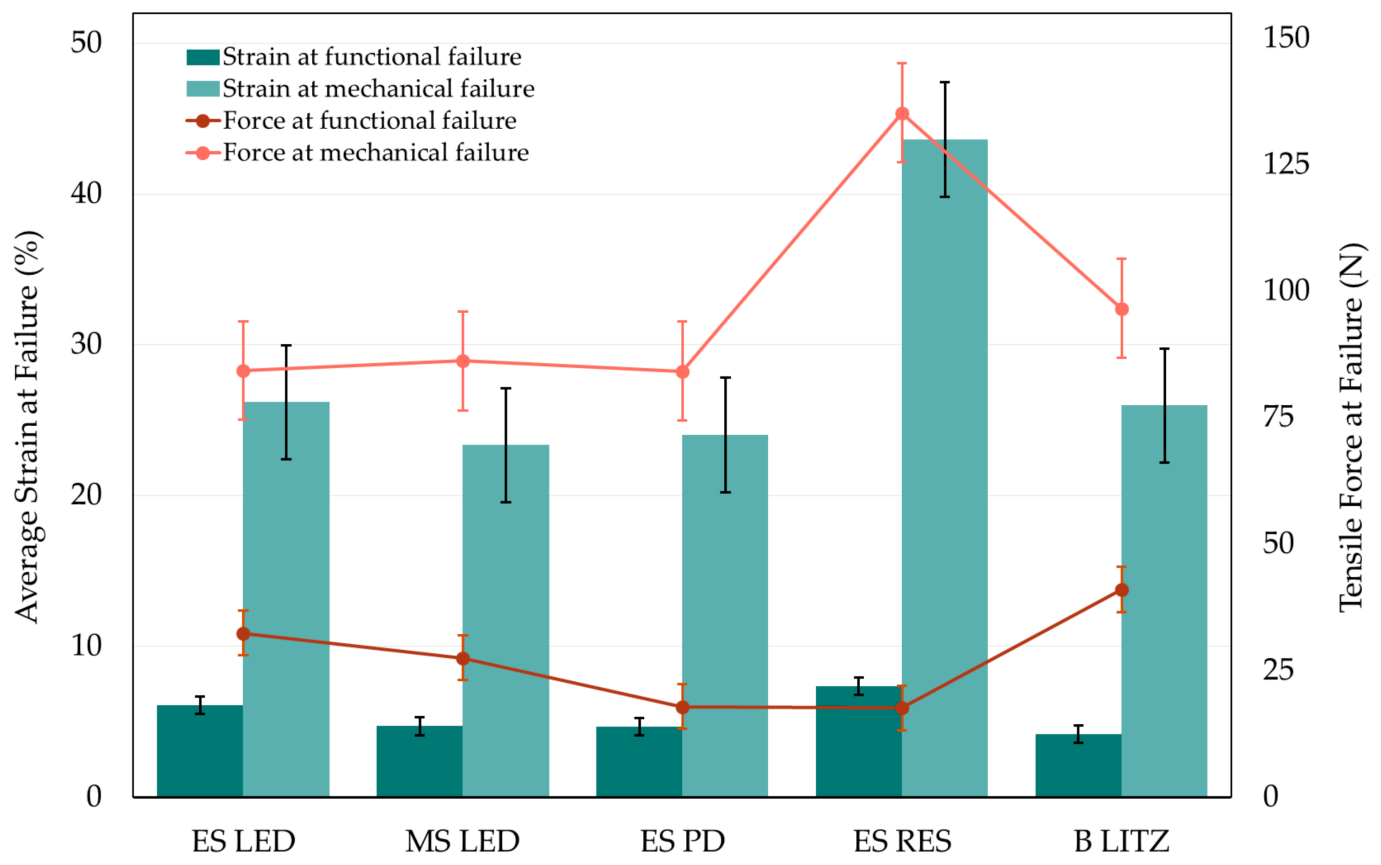
Tensile performance of the different E-yarn categories, presenting average strain at functional and mechanical failure and the corresponding tensile forces (connecting lines are for visual comparison only and do not represent a trend). Error bars indicate standard deviation.

The responses of the different E-yarn configurations to quasi-static tensile loading are presented in Table 6, with corresponding trends in deformation and load at failure illustrated in Figure 21. The values reported represent averaged results for each E-yarn configuration.

Across all configurations, two distinct thresholds were consistently observed under quasi-static tensile loading, corresponding to functional failure, defined by the loss of electrical continuity, and mechanical failure, associated with rupture of the braided textile yarn structure. For all E-yarn types, mechanical failure occurred at substantially higher strain percentage and force levels compared to functional failure. Electrical discontinuity therefore preceded structural rupture in every case. This behaviour reflects the greater sensitivity of the internal conductive pathway to deformation compared to the surrounding textile braid, which continues to sustain load beyond the point of functional failure.

Across the different yarn configurations, electrical failure generally occurred within a relatively narrow strain range. However, systematic variations were evident depending on both wiring configuration and encapsulation geometry. LED and PD E-yarns exhibited functional failure typically within the range of approximately 4–6% strain, while braided Litz samples showed a comparable value of approximately 4% strain. This may indicate a reinforcement effect associated with the polymer micro-pod, consistent with previous simulations showing that strain concentrations can be reduced by the presence of the polymer micro-pod [44].

A clear difference was observed when comparing MS LED and ES LED E-yarns, with MS LED samples exhibiting earlier functional failure. In the MS configuration, the embedded component is located within the central region of the gauge length, such that tensile loading is applied from both ends and converges toward the same location. As a result, deformation is imposed more directly on the central region, with limited opportunity for redistribution through the surrounding yarn structure. This leads to a more rapid accumulation of strain in the functional segment and consequently earlier functional failure. In contrast, in the ES configuration, the functional region is located closer to one end, allowing deformation to be initially accommodated within the surrounding textile structure before being fully imposed on the embedded segment. Hence, this configuration allows progressive engagement and redistribution of load within the yarn constituents, leading to comparatively higher strain tolerance prior to functional failure. This trend has been observed previously for another E-yarn design [7].

A further distinction is observed between the ES LED and ES PD E-yarns, where the primary difference lies in the encapsulation pod width. The slightly larger encapsulation of the ES PD E-yarns modifies the local deformation response. The thicker encapsulated region behaves as a locally stiffer inclusion within the otherwise compliant polymer–textile structure, limiting deformation within this region and causing neighbouring segments to accommodate a greater proportion of the applied strain. This leads to uneven strain distribution along the yarn, with higher strain concentrated in the adjacent regions, ultimately causing earlier functional failure under tensile loading compared to ES LED E-yarns.

In contrast, ES resistor E-yarns demonstrated a higher deformation tolerance, with functional failure occurring at approximately 7% strain, followed by significantly higher mechanical failure levels. This improved performance is associated with the thicker braid structure, which allows deformation to be more effectively accommodated within the surrounding textile layer before being transmitted to the internal conductive elements. The possible strain shielding effect may contribute to the increased electrical failure strain for the resistor-based E-yarns compared to configurations with thinner braid coverage. This behaviour is reflected consistently in Figure 21, where the separation between electrical and mechanical failure strains is significantly larger for the resistor configuration.

While similar trends are observed in both deformation and load responses, variations between yarn types reflect the heterogeneous, non-consolidated nature of the E-yarn architecture. The loosely coupled constituents enable redistribution of deformation within the structure, particularly beyond the point of electrical failure, where the textile braid continues to carry load. Consequently, the observed behaviour reflects the combined influence of internal conductive elements and the surrounding polymer–textile structure.

Overall, the results indicate that functional failure is governed by the deformation limits of the internal conductive assembly, whereas mechanical failure reflects the load-bearing capacity of the outer braided structure, with variations across yarn categories strongly influenced by wiring configuration, encapsulation geometry, and braid thickness.

To complement the observed tensile behaviour, a statistical evaluation of the tensile strain percentage at functional and mechanical failure was conducted to analyse the differences among yarn configurations. The outcomes summarised in Table 7 indicate that most comparisons were not statistically significant across the investigated configurations, with relatively high *p*-values obtained for both functional and mechanical failure. In particular, the comparison between ES and MS LED E-yarns does not reveal a distinguishable difference in tensile performance, despite the trends discussed earlier. Similarly, differences between ES LED and ES PD E-yarns remain limited. In contrast, resistor-based E-yarns exhibited a distinct behaviour at mechanical failure, showing significantly higher strain and force values compared to ES LED E-yarns, as reflected by the lower *p*-values (<0.05).

Overall, the statistical results support the earlier observations by confirming that functional failure characteristics remain broadly consistent across E-yarn configurations, while differences in mechanical failure are more pronounced for yarns with thicker braid covering structures, such as the ES resistor yarn configuration.

**Figure 22:**
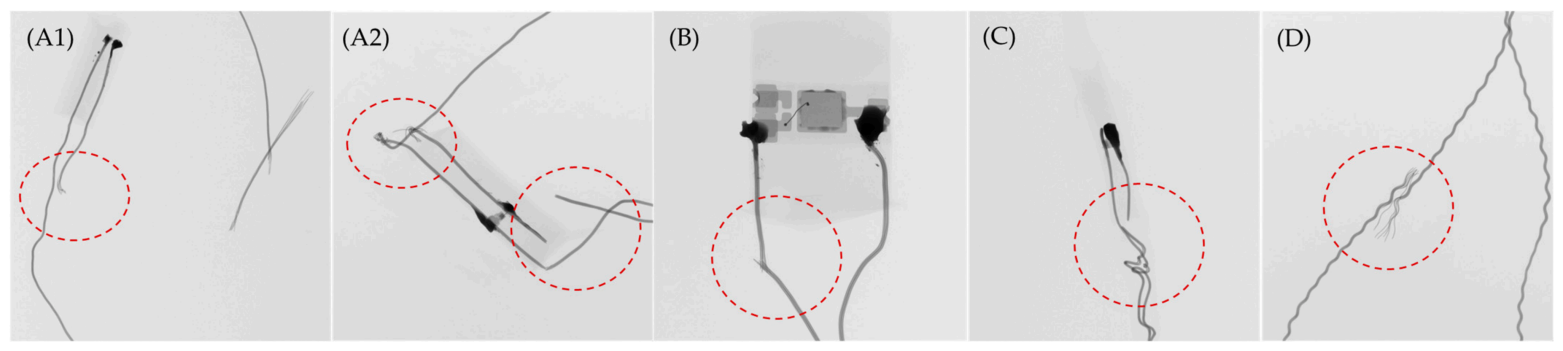
X-ray images of different E-yarn types after failure under quasi-static tensile loading, with failure locations indicated by red circles in each. (**A1**) End-soldered LED E-yarn. (**A2**) Mid-soldered LED E-yarn. (**B**) End-soldered PD E-yarn. (**C**) End-soldered resistor E-yarn. (**D**) Braided Litz wires.

X-ray imaging of samples subjected to quasi-static tensile loading revealed distinct failure features compared to those observed under cyclic conditions. Post-tensile analysis showed that failure remained primarily associated with the Litz wire conductors, although the exact fracture location varied across samples, occurring within the encapsulated region, at the encapsulation interface, and in some cases, beyond the encapsulated zone, as shown in Figure 22. This variation reflects the distribution of deformation along the yarn during tensile loading.

**Figure 23:**
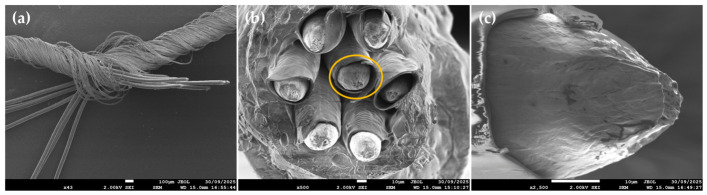
SEM images of fractured E-yarns following quasi-static tensile loading at increasing magnifications. (**a**) Fractured Litz wire region showing strand separation and elongation; (**b**) fractured Cu strand ends, with the highlighted region indicating micro-void formation; (**c**) higher-magnification view showing necking and tapered fracture morphology, consistent with ductile failure behaviour.

SEM observations of the fractured Cu strands revealed clear ductile features, including necking, filament thinning, and micro-void formation along the fracture surfaces shown in Figure 23. These characteristics are consistent with progressive plastic deformation prior to rupture, indicating a fundamentally different failure mechanism compared to bending fatigue. The presence of micro-void coalescence further confirms that fracture under tensile loading is governed by gradual material deformation and localised elongation, rather than abrupt failure.

### 3.4. Wash Durability Behaviour

**Figure 24:**
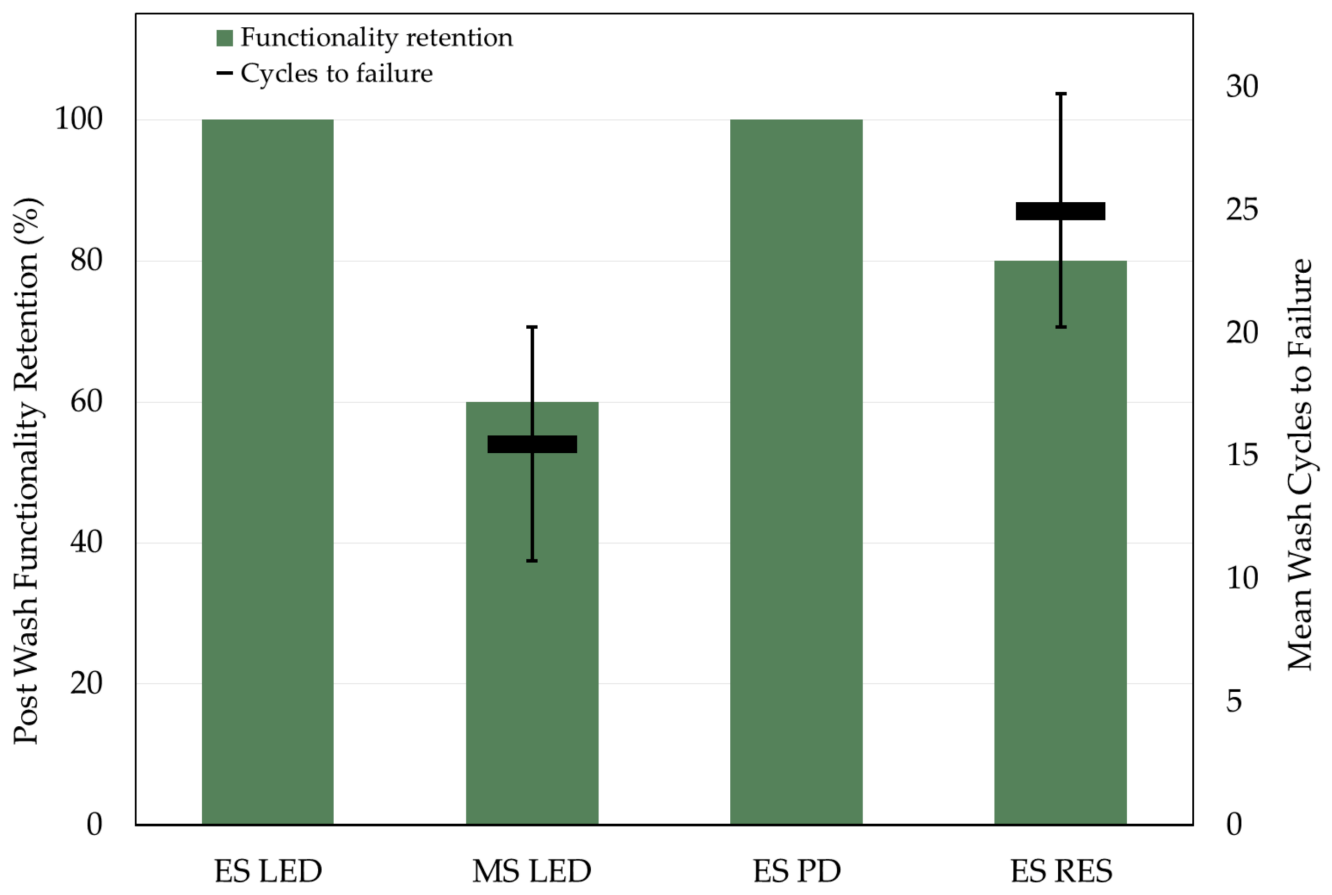
Post-wash performance of E-yarns. Functionality retention of each E-yarn configuration is presented with the bar chart and mean wash cycles to failure are indicated by the markers, with error bars representing the standard deviation.

The wash durability results indicate differences in functionality retention among the component-embedded E-yarn configurations, as illustrated in Figure 24 and summarised in Table 8, with the individual electrical response profiles provided in Appendix B.

ES LED and ES PD E-yarns retained full functionality throughout the 25 wash cycles. In contrast, the MS LED configuration showed reduced post-wash functionality retention, with two out of five samples classified as failed according to the defined functionality criterion detailed in Section 2.2.5. In both failed MS LED samples, loss of continuity occurred at only one side of the yarn, while the opposite side remained functional. ES RES also showed slightly lower functionality retention than the other ES E-yarns, with one sample failing out of five.

**Figure 25:**
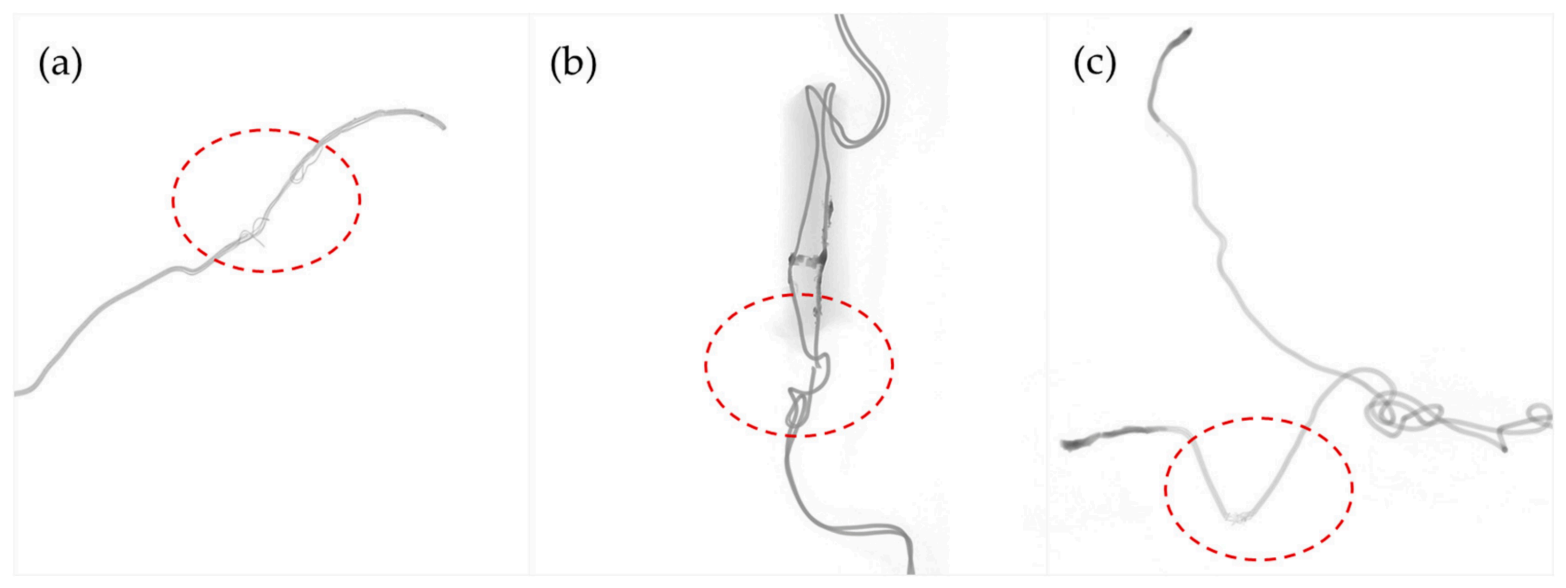
Post-wash X-ray images of E-yarns with failure locations circled in each. (**a**) Broken Litz wire end of the MS LED yarn; (**b**) fracture in Litz wire near the encapsulation region of the MS LED yarn; (**c**) broken Litz wire end of the ES RES yarn.

Post-wash structural analysis using X-ray imaging provided further insight into the failure locations. In two of the three failed samples (one MS LED and one ES RES), the failures were found to originate from breakages located at the exposed ends of the Litz wires, outside the braided E-yarn region, as shown in Figure 25a,c. These breakages were not detectable by visual inspection and were identified only through microscopic examination, with representative images of the broken exposed wire ends provided in Appendix B. In practice, in an E-textile platform incorporating E-yarns, the exposed Litz wire ends used for laboratory electrical connection would normally be terminated through robust connectors or interconnection points, rather than remaining as unsupported open wire ends. Thus, the damaged ends of the Litz wires in these two E-yarns were removed to determine whether additional failure points were present within the E-yarn structure which might not have otherwise been identified. Following removal of these damaged end sections and re-evaluation, functionality was restored in these two samples (one MS LED and one ES RES). Consequently, although two MS LED samples were initially classified as functionally failed, only one MS LED sample exhibited damage localised near the encapsulated region and was therefore considered a confirmed functional failure of the E-yarn structure, as shown in Figure 25b. This confirmed failure interpretation is also included in Table 8 to distinguish apparent electrical failures from failures associated with the E-yarn architecture itself.

This distinction highlights the importance of combining electrical functionality assessment with structural inspection when evaluating wash durability. Electrical testing alone identified loss of continuity, but X-ray and microscopic analysis were required to distinguish failures occurring at exposed wire ends from failures associated with the encapsulated component region. The comparable wash performance of the ES LED and ES PD E-yarns, despite differences in component dimensions and encapsulation pod geometry, suggests that encapsulation geometry did not have a clear influence on wash durability within the present dataset. The reduced initial retention observed for the MS LED configuration may be associated with its wiring arrangement, where conductive paths extend to both ends of the yarn. However, given that only one confirmed failure was located near the encapsulated region, this should be interpreted cautiously.

Statistical comparison of wash cycles to failure was not performed, as confirmed E-yarn failure occurred only in the MS LED configuration, while the other E-yarn categories showed no confirmed failures within the test duration. Therefore, no independent groups with comparable failure data were available for a robust cycle-based statistical comparison.

**Figure 26:**
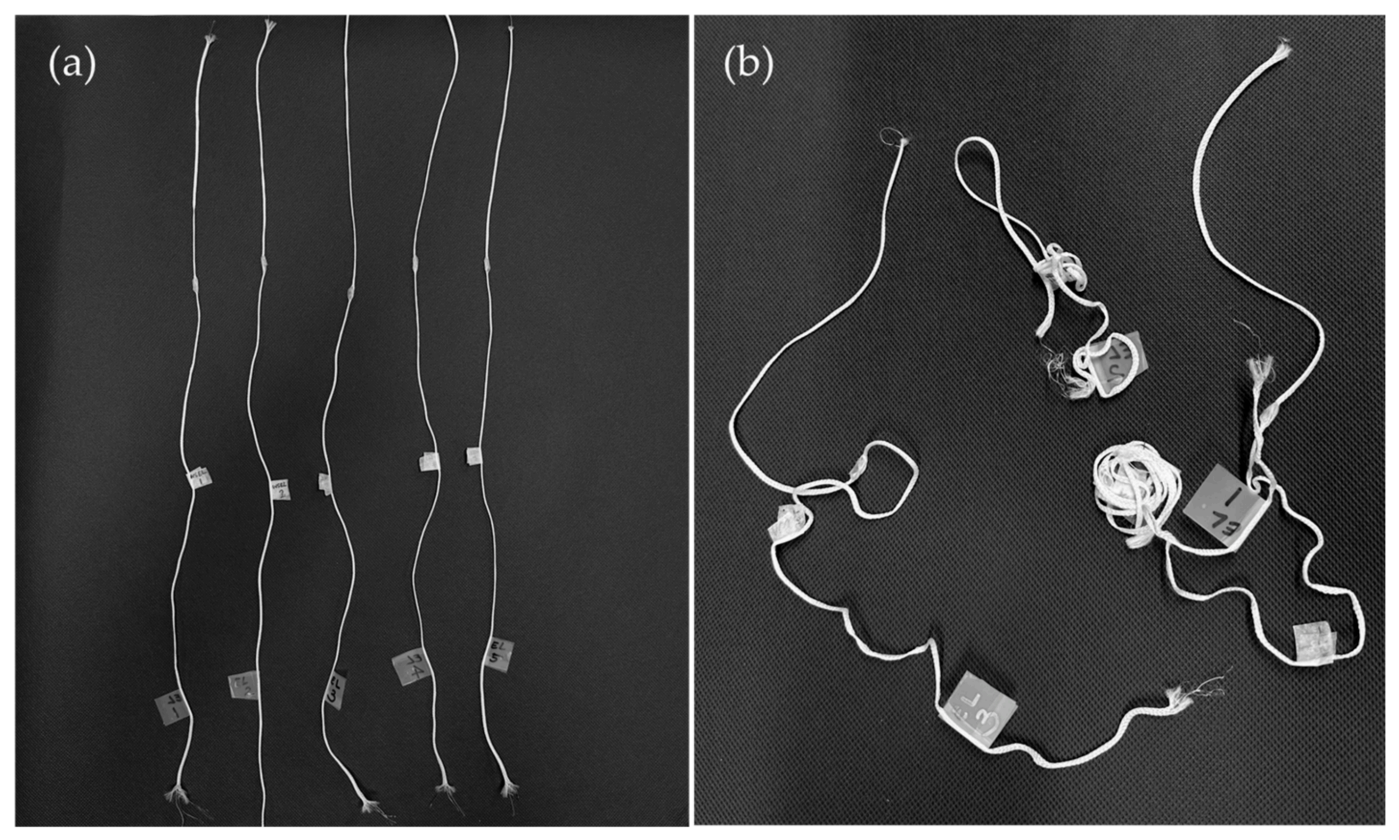
Representative E-yarn geometry before and after washing and drying, shown using ES LED samples. Similar crimping and curvature behaviour was observed across the tested categories. (**a**) Before washing; (**b**) after washing and drying.

Visual inspection of the samples before and after washing and drying further revealed a noticeable increase in yarn crimping and curvature after laundering, as presented in Figure 26. This observation indicated that the washing process induced permanent or semi-permanent geometric rearrangement of the yarns, which may alter how deformation is distributed during subsequent wash cycles. Although such crimping did not necessarily result in immediate functional failure, it highlights the cumulative mechanical effect of washing on E-yarn geometry and provides a qualitative indication of deformation history.

Overall, the wash durability results highlight that, unlike controlled mechanical loading, the washing environment imposes complex multiaxial deformation. The findings suggest that wiring configuration, exposed wire regions, and local encapsulation transitions should be considered when assessing functional stability under laundering.

The durability results could be interpreted in the context of the wide variation in E-textile testing protocols reported in the literature, where differences in bending geometry, cycle limits, wash protocols, textile integration methods and failure criteria limit direct quantitative comparison [23,28,37]. The wash durability findings are broadly consistent with previous E-yarn studies showing that some E-yarn architectures can survive repeated laundering, while failures are commonly associated with wire damage, micro-pod size and textile integration methods [9,33]. In the present study, testing was performed on standalone polymer-encapsulated E-yarns, allowing intrinsic yarn-level failure features, including conductor fracture near encapsulation transitions, wiring-configuration effects, braid-related load sharing and pod morphology variation, to be examined before textile integration.

## 4. Conclusions

This study evaluated the mechanical and functional durability of polymer-encapsulated E-yarns under different deformation modes, including cyclic bending, cyclic torsion and quasi-static tensile loading and laundering, with all tests conducted at the standalone yarn level. By combining electrical monitoring with X-ray imaging, SEM analysis, and simplified finite element modelling, the study distinguished functional failure from structural damage and provided a clearer systematic approach to understanding yarn-based electronic textile reliability.

The results showed that E-yarn durability was strongly dependent on the applied deformation mode. Cyclic bending was the most critical loading condition within the investigated test regime, producing functional failure in several E-yarn configurations. In contrast, torsional fatigue did not produce electrical or mechanical failure within the applied test limits, indicating that the tested E-yarn structures were more tolerant of twisting than of repeated bending deformation. Under quasi-static tensile loading, functional failure consistently occurred before mechanical rupture, confirming that the internal conductive pathway was more sensitive to deformation than the surrounding braided textile structure.

The bending fatigue results indicated that wiring configuration and local geometry influence durability, although statistical analysis showed that differences in cycles to failure between failed E-yarn categories were not significant within the present dataset. The MS LED configuration showed the highest bending functionality retention, suggesting that continuous wire routing and a more balanced structural arrangement may improve resistance to cyclic bending. However, the results also showed that failure behaviour cannot be attributed to a single design factor alone, as encapsulation geometry, braid structure, wire arrangement, and manufacturing variation may all contribute to the observed response.

Finite element modelling provided a simplified numerical assessment of stress and strain distribution during bending. The simulations showed increased local mechanical loading in the bent state near the imposed bending region close to the encapsulation exit. However, the FEM-derived stress and strain ranking did not directly align with the experimental fatigue performance, particularly for the MS LED configuration. Therefore, the model can be interpreted as a tool for understanding local stress and strain distribution under the selected bending setup, rather than as a quantitative predictor of fatigue life.

Post-failure X-ray and SEM analyses provided important evidence for the underlying failure mechanisms. Under cyclic bending, fractures were mainly located in the braided Litz wire region near the encapsulation transition, while SEM observations showed relatively flat fracture surfaces without visible necking, consistent with brittle, fatigue-driven Cu strand fracture. In contrast, tensile loading produced ductile fracture features, including necking, filament thinning, and micro-void formation. These findings confirm that the same E-yarn architecture can fail through different mechanisms depending on the imposed loading condition.

Quasi-static tensile testing showed two distinct failure stages across all E-yarn categories: functional failure, associated with loss of electrical continuity, and mechanical failure, associated with rupture of the braided textile yarn structure. Functional failure consistently occurred before mechanical rupture, confirming that the internal conductive pathway was more sensitive to tensile deformation than the surrounding braided structure. Statistical analysis showed that functional failure behaviour was broadly similar across the investigated configurations, while mechanical failure behaviour was more distinct for the resistor-based E-yarns, which exhibited higher strain and force values at mechanical failure. This suggests that braid structure contributes more clearly to load-bearing behaviour under tensile loading than to bending fatigue resistance.

Wash durability testing showed generally high functionality retention among the component-embedded E-yarn configurations. Post-wash structural analysis indicated that some apparent failures originated from breakages at the exposed ends of the Litz wires outside the braided region, rather than from the encapsulated functional region. After removal of these damaged end sections, functionality was restored in selected samples. Only one MS LED sample exhibited true functional failure associated with the encapsulated region. These results show that electrical functionality assessment should be supported by structural analysis to identify the true failure location after laundering.

Overall, the findings show that the functional reliability of polymer-encapsulated E-yarns is governed by the combined effects of loading mode, wiring configuration, encapsulation geometry, braid structure, local transition regions and fabrication variability. The transition between the encapsulated and non-encapsulated yarn regions was identified as a mechanically sensitive location under cyclic bending. The statistical analysis provides a more robust basis for interpreting the observed trends, showing that some apparent differences in failure incidence do not necessarily correspond to statistically distinct fatigue lives. This highlights the importance of combining cyclic bending, torsional fatigue, tensile loading, wash durability, electrical monitoring, and structural failure analysis when assessing E-yarn reliability.

A key limitation of this study lies in the coupled nature of the E-yarn architecture, where the effects of encapsulation and braid structure on mechanical and electromechanical performance could not be fully decoupled. This restricts the ability to isolate the contribution of individual structural components to the observed behaviour. These limitations are primarily attributed to the practical constraints of time and experimental scope, but addressing them will be essential for developing a more complete understanding of durability in heterogeneous E-yarn systems.

Future work should isolate the effects of braid structure by systematically varying braiding materials and fabrication parameters while incorporating more detailed multistrand and textile-inclusive FEMs. Longer-term cyclic testing, combined deformation modes, and improved monitoring of functional evolution would further support the development of more reliable E-yarns for wearable electronic textile applications.

## Figures and Tables

**Table 1:** Summary of E-yarn configuration specifications and fabrication parameters. Dimensions and structural characteristics are provided for each configuration to enable comparison of design variables.

E-Yarn Configuration	Encapsulation Pod Dimensions	Encapsulation Pod Shape	Covering Yarn	Yarn Carriers	Lay Length	E-Yarn Thickness
End-soldered LED (ES LED)	2 mm × 2 mm × 5 mm	Cuboid	Polyester (1 × 1/167/48)	12	10 mm	1.26 ± 0.03 mm
Mid-soldered LED (MS LED)	2 mm × 2 mm × 5 mm	Cuboid	Polyester (1 × 1/167/48)	12	10 mm	1.25 ± 0.05 mm
End-soldered PD (ES PD)	4 mm × 2 mm × 5 mm	Cuboid	Polyester (1 × 1/167/48)	12	10 mm	1.29 ± 0.02 mm
End-soldered Resistor (ES RES)	1.5 mm (φ) × 5 mm	Cylindrical	Polyester (2 × 1/167/36)	24	6 mm	1.79 ± 0.06 mm
Braided Litz (B LITZ)	-	-	Polyester (1 × 1/167/48)	12	10 mm	1.24 ± 0.04 mm

**Table 2:** Bending fatigue performance of E-yarn configurations, showing failure percentage at specified cycle limits and corresponding mean cycles to failure under different testing conditions.

E-Yarn Configurations	Bending Frequency	Bending Cycle Limit	Bending Failure (at the Cycle Limit)	Bending Cycles to Failure (Mean ± SD)
ES LED	1 Hz	2500	60%	1433.33 ± 623.6
	0.5 Hz	2500	60%	666.7 ± 205.5
MS LED	1 Hz	2500	0%	No failure
ES PD	1 Hz	2500	20%	1700.00 ± 0
ES RES	1 Hz	2500	60%	1334 ± 48.1
B LITZ	1 Hz	2500	70%	1304.4 ± 625
ES RES	1 Hz	10,000	80%	3382.75 ± 3548.78
B LITZ	1 Hz	10,000	100%	2114.00 ± 1588.98

The shaded rows indicate the ES RES and B LITZ configurations subjected to extended bending fatigue testing up to 10,000 cycles while all other configurations were evaluated within the 2500-cycle limit.

**Table 3:** Maximum equivalent stress and strain values obtained from the FEMs during bending deformation from the recovery state to the bent state.

E-Yarn Configurations	Maximum Stress (Recovery State)	Maximum Stress (Bent State)	Maximum Strain (Recovery State)	Maximum Strain (Bent State)
ES LED	381.45 MPa	466.43 MPa	0.348%	0.428%
MS LED	355.18 MPa	469.58 MPa	0.324%	0.430%
ES PD	377.34 MPa	438.81 MPa	0.345%	0.407%
ES RES	336.05 MPa	394.88 MPa	0.316%	0.367%

**Table 4:** Statistical comparisons of cycles to failure between selected E-yarn configurations and test conditions. Each comparison isolates a single primary factor, either a design parameter or a test condition. Degrees of freedom (df), t-values, and corresponding *p*-values are presented.

Comparison	Primary Factor	df	t-Value	*p*-Value
ES LED vs. ES PD	Encapsulation geometry	5.02	0.39	0.71
ES LED vs ES RES	Braid structure	5	−1.05	0.34
ES LED vs. B LITZ	Component presence	11.87	0.40	0.69
1 Hz vs. 0.5 Hz	Bending frequency	6.83	2.44	0.045

The shaded row compares ES LED samples tested at bending frequencies of 1 Hz and 0.5 Hz, while all other rows compare different E-yarn configurations tested at 1 Hz.

**Table 5:** Torsional fatigue performance of E-yarn configurations with the test conditions and test limits.

E-yarn Configuration	Torsional Frequency	Torsion Cycles	Torsional Failure
ES LED	0.5 Hz	2500	0%
ES PD	0.5 Hz	2500	0%
ES RES	0.5 Hz	10,000	0%
B LITZ	0.5 Hz	10,000	0%

**Table 6:** Average strain percentage and tensile force at functionality and mechanical failure across E-yarn categories.

E-Yarn Configuration	Average Strain Percentage at Failure (%)		Average Tensile Force at Failure (N)	
	Functional Failure	Mechanical Failure	Functional Failure	Mechanical Failure
ES LED	6.09 ± 4.12	26.18 ± 3.61	32.47 ± 14.97	84.29 ± 8.77
MS LED	4.70 ± 0.43	23.33 ± 3.32	27.56 ± 10.27	86.24 ± 7.15
ES PD	4.70 ± 0.46	24.00 ± 2.31	17.93 ± 12.06	84.21 ± 7.60
ES RES	7.38 ± 1.20	43.61 ± 9.41	17.64 ± 10.91	135.30 ± 25.53
B LITZ	4.20 ± 0.59	25.96 ± 0.93	41.08 ± 5.94	96.62 ± 0.72

**Table 7:** Statistical comparison of tensile strain percentage at functional and mechanical failure across E-yarn configurations, including degrees of freedom (df), t-values and *p*-values.

Comparison	Primary Factor	Functional Failure			Mechanical Failure		
		df	t-Value	*p*-Value	df	t-Value	*p*-Value
ES LED vs. MS LED	Wiring configuration	4.27	0.75	0.50	7.96	1.30	0.23
ES LED vs. ES PD	Encapsulation geometry	4.30	0.74	0.50	7.04	1.11	0.30
ES LED vs. ES RES	Braid structure	5.55	−0.66	0.53	5.04	−3.98	0.01
ES LED vs. B LITZ	Component presence	5.89	1.00	0.35	5.02	0.11	0.91

**Table 8:** Summary of wash durability performance, showing failure percentage and cycles to failure, pre- and post-structural analysis using X-ray imaging.

E-Yarn Configuration	Wash Cycles	Failure Percentage	Cycles to Failure (Mean ± SD)	Failure Percentage (Post-Structural Analysis)	Cycles to Failure (Post-Structural Analysis)
ES LED	25	0%	No failure	0%	No failure
MS LED	25	40%	15.5 ± 8.5	20%	24 ± 0
ES PD	25	0%	No failure	0%	No failure
ES RES	25	20%	25 ± 0	0%	No failure

The shaded columns report confirmed E-yarn failures and corresponding cycles to failure following post-wash structural analysis.

**Figure A1:**
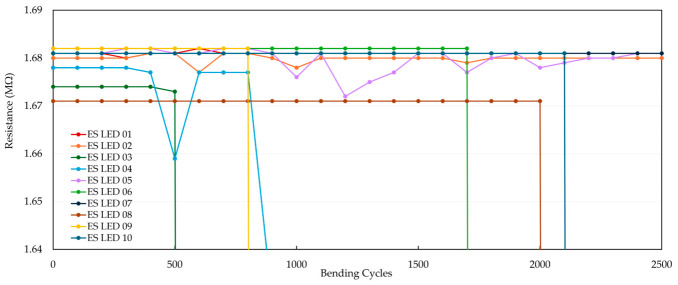
Resistance variation in end-soldered LED samples in overbending cycles at a bending frequency of 1 Hz. Electrical measurements were recorded at 100-cycle intervals.

**Figure A2:**
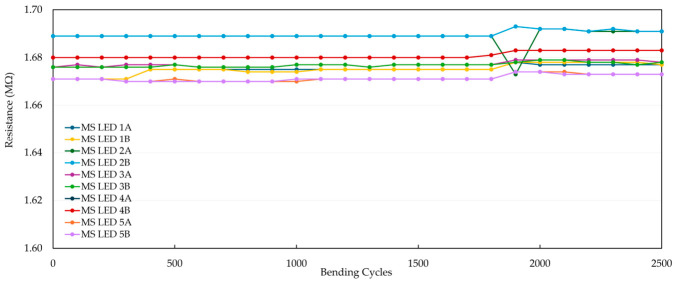
Resistance variation in mid-soldered LED samples in overbending cycles at a bending frequency of 1 Hz. Electrical measurements were recorded at 100-cycle intervals.

**Figure A3:**
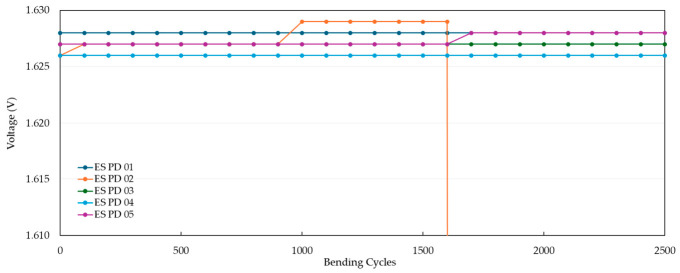
Voltage variation in end-soldered PD samples in overbending cycles at a bending frequency of 1 Hz. Electrical measurements were recorded at 100-cycle intervals.

**Figure A4:**
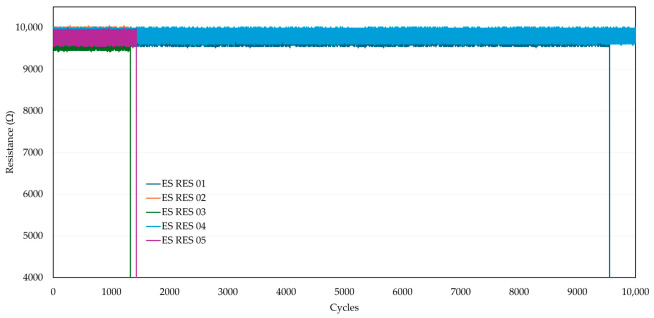
Resistance variation in end-soldered RES samples in overbending cycles at a bending frequency of 1 Hz. Electrical measurements were recorded continuously at 40 ms intervals.

**Figure A5:**
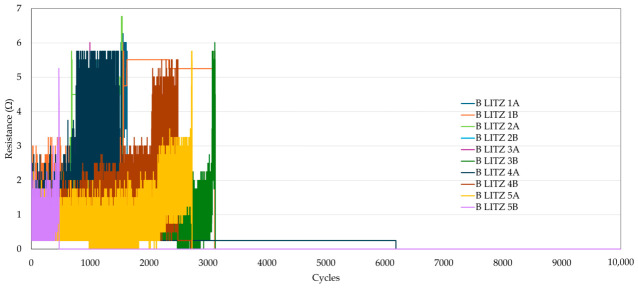
Resistance variation in braided Litz samples in overbending cycles at a bending frequency of 1 Hz. Electrical measurements were recorded continuously at 40 ms intervals.

**Figure A6:**
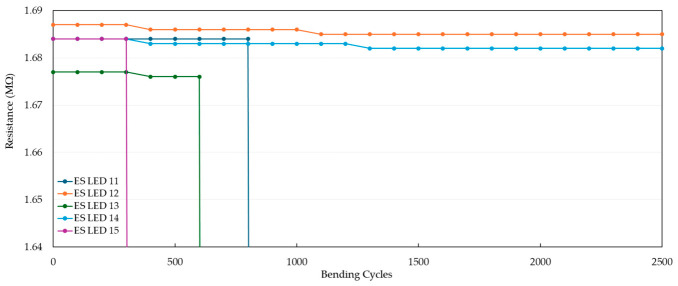
Resistance variation in end-soldered LED samples in overbending cycles at a bending frequency of 0.5 Hz. Electrical measurements were recorded at 100-cycle intervals.

**Figure A8:**
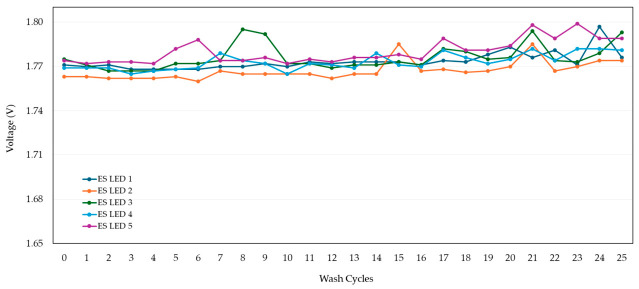
Voltage variation in end-soldered LED samples across the wash cycles.

**Figure A9:**
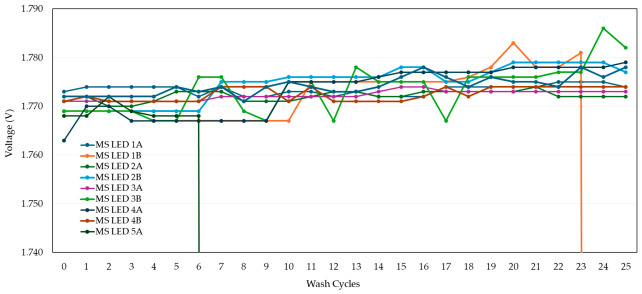
Voltage variation in mid-soldered LED samples across the wash cycles.

**Figure A10:**
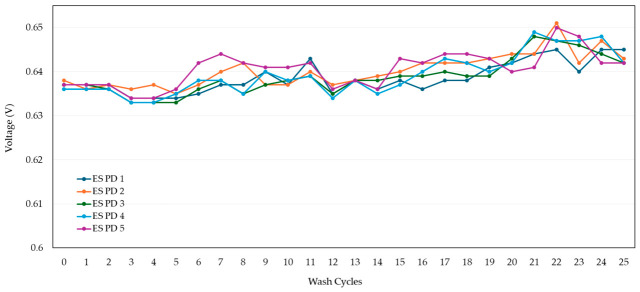
Voltage variation in end-soldered PD samples across the wash cycles.

**Figure A11:**
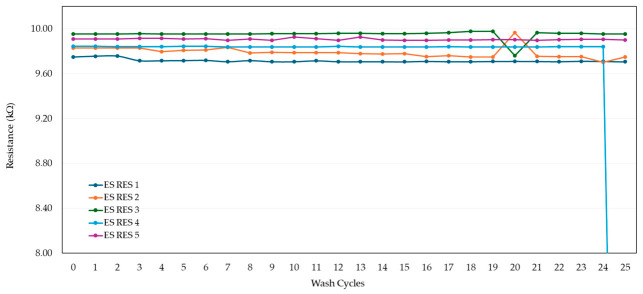
Resistance variation in end-soldered resistor samples across the wash cycles.

**Figure A12:**
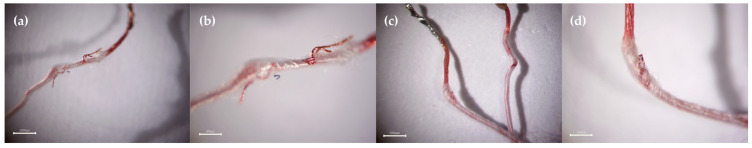
Microscopic images of broken exposed Litz wire ends located outside braided structure, in wash test failed samples. (**a**,**b**) MS LED sample shown at different magnifications; (**c**,**d**) ES RES sample shown at different magnifications.

## Data Availability

Data are contained within the article. All data, graphs and images presented in this paper are openly available at https://doi.org/10.6084/m9.figshare.32477466 (accessed on 1 August 2026).

## Supplementary Materials

The following supporting information can be downloaded at: https://www.mdpi.com/article/10.3390/polym18151923/s1, Video S1: FEM Simulation of Bending Deformation.

## Abbreviations

The following abbreviations are used in this manuscript:

E-yarn	Electronic yarn
E-textile	Electronic textile
ES	End soldered
MS	Mid soldered
LED	Light Emitting Diode
PD	Photodiode
RES	Resistor
B LITZ	Braided Litz
IMU	Inertial Measurement Unit
cpm	Cycles per minute
SEM	Scanning Electron Microscopy
SD	Standard Deviation
df	Degrees of Freedom
GSM	Grams per Square Metre
RH	Relative Humidity

## Appendix A

This appendix presents the individual electrical measurements recorded during cyclic bending fatigue testing for the E-yarn configurations tested under the specified bending conditions. It also summarises the distribution of encapsulation micro-pod termination morphologies and the corresponding failure incidence within the bending fatigue test subset. The complete underlying datasets corresponding to these graphical representations are provided in the data archive.

**Table A1:** Summary of sample count and percentage distribution of encapsulation micro-pod termination morphologies of E-yarn configurations.

E-Yarn Configuration	Total Samples	Sample Count			Percentage of Samples		
		Conical	Straight	Not Clearly Visible	Conical	Straight	Not Clearly Visible
ES LED	35	17	14	4	48.6%	40.0%	11.4%
MS LED	20	14	5	1	70.0%	25.0%	5.0%
ES PD	20	7	9	4	35.0%	45.0%	20.0%

**Table A2:** Summary of sample count and percentage incidence of conical pod termination morphology and associated bending failure within the bending fatigue test subset.

E-Yarn Configuration	Total Sample Count	Conical Shaped Sample Count	Conical Shaped Failed Sample Count	Conical Shape Presence	Failure Percentage with Conical Shape
ES LED	15	9	6	60.0%	67%
MS LED	5	2	0	40.0%	0
ES PD	5	1	0	20.0%	0

## Appendix B

This appendix presents the individual electrical measurements recorded after each wash cycle, during wash durability testing for the component-embedded E-yarn configurations, together with microscopic images of failed samples showing damage to exposed Litz wire ends outside the braided structure.
